# Integrating natural variation through GWAS – genetics of drought and flood tolerance in grass pea reveal independent yet interconnected mechanisms

**DOI:** 10.1186/s12870-026-08229-y

**Published:** 2026-02-05

**Authors:** Matilde Sanches, Marnik Vuylsteke, Carmen Santos, Amna Mhamdi, Susana Araújo, Frank Van Breusegem, Maria Carlota Vaz Patto

**Affiliations:** 1 Instituto de Tecnologia Química e Biológica António Xavier, ITQB NOVA, Universidade Nova de Lisboa, Oeiras, 2780-157 Portugal; 2 Department of Plant Biotechnology and Bioinformatics, Ghent University, Ghent, 9052 Belgium; 3 Center of Plant Systems Biology, VIB, Ghent, 9052 Belgium; 4 Gnomixx, Melle, 9090 Belgium; 5 MORE – Laboratório Colaborativo Montanhas de Investigação, Bragança, Portugal; 6 CIMO, LA SusTEC, Instituto Politécnico de Bragança, Campus de Santa Apolónia, Bragança, 5300-253 Portugal

**Keywords:** *Lathyrus sativus*, GWAS, Water deficit, Waterlogging, Genotype–phenotype association

## Abstract

Grass pea (*Lathyrus sativus* L.) is a grain legume of increasing importance in the Mediterranean region due to its outstanding tolerance to abiotic stresses such as salinity, heat, drought, and flooding, outperforming many other legume species. Despite established natural phenotypic variation in response to water-related stresses, the genetic basis of this resilience remains poorly understood, hindering precision breeding for single and combined stress tolerance. A genome-wide association study was conducted here to investigate the genetic architecture of water stress responses in grass pea. Previously, phenotypic data, including gas exchange, chlorophyll *a* fluorescence, photosynthetic pigments, leaf water status, and biomass partitioning traits, were assessed under well-watered, mild drought, and partial submergence conditions across 194 representative grass pea accessions worldwide. The data were associated with 5,651 single nucleotide polymorphisms (SNPs) using linear mixed models under a restricted maximum likelihood framework, incorporating population structure and the newly assembled L0007 genome. A total of 130 unique SNPs associated with at least one trait-treatment combination or with trait variation between stress and control conditions, providing a valuable resource for precision breeding of multi-stress tolerance in grass pea. The loci associated with drought and waterlogging were largely non-overlapping, suggesting distinct genetic bases for the two stress tolerances. However, some common mechanisms, such as redox regulation and carbohydrate metabolism, emerged among the identified candidate genes, highlighting some interconnectedness of biological pathways involved in grass pea responses to water stress.

**Supplementary Information:**

The online version contains supplementary material available at 10.1186/s12870-026-08229-y.

## Key message

Genotypic-phenotypic data associations suggest that the documented tolerance to water deficit and to waterlogging in grass pea is achieved through independent molecular mechanisms that might nevertheless overlap.

**Supplementary Information:**

The online version contains supplementary material available at 10.1186/s12870-026-08229-y.

## Introduction

*Lathyrus sativus* L., commonly known as grass pea, is an annual, primarily self-pollinating grain legume recognized for its outstanding tolerance to adverse conditions, such as water stress (defined here as water deficit and waterlogging), salinity, and temperature extremes [5, 19, 55, 98]. Not only is grass pea an insurance crop in some of the world’s most vulnerable regions, it also holds huge potential as a genetic resource for breeding climate-resilient varieties [30].

Grass pea’s tolerance to drought is partially explained by its morphology, including narrow leaves, winged stem margins, and a deep, extensive root system [48], as well as with a high photosynthetic capacity and efficient water use [15, 35, 89]. Additionally, biochemical changes can also occur, such as enhanced performance of antioxidant activity, reactive oxygen species (ROS) scavenging, and effective osmotic adjustment [37, 67, 95]. However, knowledge on the molecular basis of drought resilience in grass pea remains limited, with existing studies focusing on broad changes in proteomic [20, 75] and transcriptomic profiles [37], and the identification of a few drought-responsive microRNAs [14].

Short-term waterlogging (14 days with the water table 10 mm below the soil surface) had no significant effect on shoot and root dry biomass in grass pea [55]. This resilience has been attributed to increased antioxidant activity as well as the formation of aerenchyma and lateral roots under partial submergence [110]. Under combined stress conditions, such as heat with drought [4] or salinity with drought [95], grass pea accessions exhibited significant differences in yield-related and physiological traits, including osmotic adjustment and β-N-oxalyl-l-α,β-diamino propionic acid (β-ODAP) accumulation.

In addition to the aforementioned variation among individual accessions, evidence suggests that grass pea exhibits distinct responses to water availability based on geographical origin and seed type—the main clustering parameters in this species [68]. For example, seed germination in waterlogged soils varies by geographical origin, with accessions from Bangladesh and Ethiopia being the most well adapted [105]. Under Turkish rainfed field conditions, subtropical accessions consistently outperformed East European accessions across multiple yield parameters, regardless of the water availability [39].

We recently conducted an extensive phenotyping study on a worldwide collection of 194 grass pea accessions, which were subjected to three distinct water regimes: well-watered (WW), water deficit (WD), and waterlogging (WL). Several physiological and morphological traits were quantified to characterize the diverse responses to contrasting water stresses [82]. Under WD, increased root growth and stomatal closure were often observed, contributing to improved water use efficiency. In contrast, enhanced shoot growth and carotenoid accumulation appeared to be the key responses to the WL treatment. Interestingly, the physiological and relative performance ranking of accessions varied across water treatments, indicating a strong genotype-treatment interaction [82]. We also demonstrated variability across ecotypes. For instance, light and large seeds (typically the Mediterranean ecotype produced enlarged plants with increased total dry biomass overall, whereas dark and small-seeded accessions (mainly of African and Asian origin seemed to be physiologically less affected by contrasting water stresses, exhibiting significantly augmented relative water content, stomatal conductance, and water use efficiency under WD,as well as improved stability or even amplified pigment content under WL [82].

Nevertheless, the molecular and genetic architecture of water stress resilience in grass pea remains largely unexplored, resulting in limited precision breeding for multi-stress tolerance. The availability of cost-effective, high-throughput platforms for single-nucleotide polymorphism (SNP) genotyping are important in prompting genetic research in underused crops such as grass pea [38]. Thus far, only a few studies have investigated the genetics of interesting traits in this species. They identified candidate genes for disease resistance [58, 59, 80, 84] and discovered a key gene in the β-ODAP biosynthesis pathway [25], but none directly assessed the genetic basis of abiotic stress tolerance. Given the complexity of these traits, influenced by multiple genes and with strong environmental interactions, genome-wide association studies (GWAS) offer a powerful and adequate approach to clarify the genetic architecture underlying such complexity [42, 64].

The identification of quantitative trait loci (QTLs) for water stress tolerance in other legumes has predominantly been achieved through GWAS, although breeding for complex traits in legumes has historically lagged behind that of cereals [78]. With regard to drought tolerance, GWAS has facilitated the characterisation of marker-trait associations and candidate genes in soybean (*Glycine max*) [2, 40, 77, 79], common bean (*Phaseolus vulgaris*) [34, 50], faba bean (*Vicia faba*) [32], and cowpea (*Vigna unguiculata*) [83, 106]. Waterlogging tolerance has also been investigated through GWAS in soybean [88], common bean [90] and mung bean (*Vigna radiata*) [45]. The wide array of gene-trait associations reported in these studies (with the exception of the mung bean study, in which a single SNP was found to be associated with five out of six traits), highlights the complex nature of drought and waterlogging stress tolerance in legumes.

Here, we utilized the natural variation of grass pea through a GWAS approach, by means of the newly released genome assembly [100], to elucidate the molecular mechanisms and genetic basis of its water stress tolerance. We combined a genotypic dataset comprising 5,651 SNP markers mapped along the seven chromosomes of this species [81], with phenotyping data from 194 accessions that were evaluated under the WW, WD, and WL treatments, as previously described [82]. The marker-trait associations identified across the different water regimes provide the first steps towards developing molecular tools to support precision breeding for multi-stress tolerance, contributing to closing the gap between fundamental genetic research and its application in sustainable crop improvement [24, 41].

## Materials and methods

### Plant material

A set of 194 *Lathyrus sativus* L. accessions representative of the worldwide diversity of grass pea was used that had previously been genotyped using high-throughput SNP markers [81] and phenotyped for disease response, adaptation to the Mediterranean environment, and tolerance to different water stresses [30, 58, 59, 80, 82]. Within this collection, 92 and 102 accessions had light-coloured and dark-coloured seeds, respectively (visually scored). Additionally, 58 and 136 accessions were classified as large and small, based on 100-seed weight, using 18 g as the threshold [80]. Geographically, the set included 87 Mediterranean, 49 South Asian, 21 East European, 15 Sub-Saharan African, 13 North and Central Asian, 2 North American, and 2 South American accessions, and 5 of unknown origin (Supplementary Table S1).

 The studied accessions were either provided by plant germplasm banks or donors, as in the case of cultivars, breeding lines or traditional landraces seeds. In this last case, the formal identification was done by Dr Susana Neves (PhD, botanist). In detail, two of the studied accessions, SITNICA and STUDENICA, are registered Serbian grass pea cultivars (obtained and provided by IFVCNS: Institute of Field and Vegetable Crops, Serbia); the accession LISA is a breeding line (obtained and provided by Dr Diego Rubiales, IAS-CSIC, Spain), as well as the accessions W6-39,226/Raipur and LS87124, provided by Prof. Fernand Lambein (IPBO, Belgium). Accessions named PTLS#### and SNVP-64 are traditional grass pea landraces provided by Portuguese farmers, which names can be found in Supplementary Table S1. Regarding these traditional landraces, vouchers have not been deposited in any publicly available herbarium, their seeds were voluntarily provided by the farmers who own and cultivate them with the support of Ana Moral, Diego Rubiales, Letice Gonçalves, Nuno Almeida, Susana Neves, and Carlota Vaz Patto. All the other studied accessions were obtained from three different plant germplasm banks (CRF-INIA: Plant Genetic Resources Centre—National Center of CSIC (Spain), ICARDA: International Center for Agricultural Research in the Dry Areas (Lebanon) and USDA: United States Department of Agriculture (USA)). All these germplasm bank accessions, as well as accession W6-39,226/Raipur are indexed at Genesys online platform (https://www.genesys-pgr.org). There, detailed information relative to its origin, identification and available vouchers can be retrieved using the accession IDs indicated in Supplementary Table S1.

### Phenotypic data

The phenotypic dataset used in the present association analysis has been previously described [82]. Briefly, 20 different physiological traits including pigment content, gas exchange rates, chlorophyll *a* fluorescence, and dry biomass-related traits (listed in Supplementary Table S2.1), were measured in 194 grass pea accessions grown under one of three water treatments for 21 days. The treatments were well-watered (WW), which maintained approximately 95% field capacity (FC); water deficit (WD), which consisted of regular watering for 16 days followed by 5 days without irrigation, reaching approximately 55% FC; and waterlogging (WL), involving well-watered conditions for 7 days, followed by 14 days of partial submergence (approximately 1 cm above soil level). For additional methodological details, we refer to Sanches et al. [82] (Supplementary Table S2).

After filtering outliers, best linear unbiased estimates (BLUEs) were calculated for each accession, water treatment, and trait through a restricted maximum likelihood procedure [82]. These BLUEs datasets, one per trait, constituted the first phenotypic input data used for the present association study. The second phenotypic input data were the calculated deltas (Δ) for each trait and accession, specifically ΔWD = BLUE_WD_—BLUE_WW_ and ΔWL = BLUE_WL_—BLUE_WW_. Finally, the eigenvalues of each accession-treatment combination in the first five principal components (PCs) of the multivariate analysis [82], integrating all the 20 measured traits, were used as the third set of phenotypic input data.

### Genotypic data

DNA had previously been isolated from one representative individual of each grass pea accession in the studied collection at the PlantX Lab and been sent to the relevant providers for genotyping using DArTseq™ and genotyping-by-sequencing (GBS) analysis [81]. A genotypic dataset consisting of 5,651 high-quality SNPs was retrieved from this previous analysis. Importantly, genotypic variation was guaranteed in the dataset, with all included markers exhibiting a minor allele frequency greater than 5%.

Of the 5,651 genotyped high-quality SNPs, 4,871 had a unique location on the seven grass pea chromosomes [100], determined by alignment with the BLASTn tool (e-value < 1e-5) with the OmicsBox v2.0 software. The remaining 780 SNPs were considered unmapped, either because no BLAST hits passed the e-value threshold, or because multiple hits occurred of identical alignment probability to different genomic regions (based on bit score, percentage of similarity, and e-values).

### Association-mapping analysis

Genome-wide association studies (GWAS) were conducted with Genstat® software [102] in the mixed model framework to detect SNPs associated with the variation of the measured traits in the grass pea collection. Markers and accessions were fitted as fixed and random terms respectively using restricted maximum likelihood [56]. A model accounting for familial relatedness (K) was employed using a kinship matrix K [Phenotype = SNP + Accession + Error], computed using the Dice similarity coefficient (DICE method) in Genstat® with structured accession random effects [56]. The effect of the minor-frequency SNP allele was estimated in relation to the most frequent reference allele.

Marker–trait associations were filtered using a false discovery rate (FDR) procedure based on the Benjamini–Hochberg method, adapted as described by Brzyski et al. [18]. We controlled the FDR at Q = 0.20, using the number of linkage disequilibrium (LD) blocks (k = 5,433) as the effective number of independent tests rather than the total number of markers. Such a threshold was chosen to allow the detection of a larger set of marker–trait associations compared with more stringent FDR approaches, which was considered preferable given the exploratory nature of this study, and particularly the limited genomic resources available for orphan crops such as grass pea, even at the cost of following up a limited number of false-positive associations.

### Favourable allele frequency analysis

Previously, differences in response to the water treatments depending on grass pea ecotype clustering parameters (seed colour, seed size, and geographical origin of the accessions) have been reported [82]. To deepen our understanding of the differences among ecotypes, we searched for differences between the frequency of the favourable allele (i.e. maximizing the trait value) of each associated marker in light- vs. dark-seeded accessions, in large- vs. light-seeded accessions, and among the seven geographical origins in the collection.

Favourable allele frequencies were calculated for each seed morphology category (dark or light, small or large) and geographical origin, by dividing the number of accessions with the favourable allele by the total number of accessions in the same category. Differences between favourable allele frequencies depending on seed colour and size were tested with penalized generalized linear mixed models for binomial data. Because accessions share ancestry and population structure, allele frequencies are correlated within seed morphology and origin groups. Population structure therefore needs to be accounted for in post-GWAS analyses. However, for binomial allele-frequency data, kinship matrices cannot be incorporated as straightforwardly as in Gaussian GWAS LMMs, since non-Gaussian GLMMs lack closed-form likelihoods and become computationally slow and difficult to diagnose. We therefore accounted for population structure in the post-GWAS GLMMs by including the first 15 principal components from a previous eigen analysis of a subset of markers as a low-rank approximation of kinship. Seed colour and seed size were therefore set as fixed factors and population structure data as random factors. A significance level of 0.05 was applied with Bonferroni correction for multiple testing within each set of same trait- and treatment-associated markers.

The genetic variance contributed by a QTL (V_QTL_) to the total variance of each phenotypic trait (V_g_) was calculated as $\frac{2\times Freq \times \left(1-Freq\right)\times {Effect}^{2}}{{s}^{2}}$ [103], where Freq is the frequency of the favourable QTL-associated allele in the population, Effect is the estimated effect of the same allele over the trait, and s^2^ is the trait variance (the variance of the BLUEs of all accessions in all treatments; Supplementary Table S2). All statistics, visual representations, and follow-up analysis were performed with R software [73].

### Candidate gene identification

Following GWAS, a gene was considered a candidate contributing to the phenotypic trait if it contained an associated SNP with that trait. Putative candidate genes were identified with the MapMan and Mercator4 v2.0 web tools [86] to locate the sequences of associated SNPs in relation to the grass pea genome. Swiss-Prot was the preferred source for annotation, although the InterPro-UniProt database was consulted as well.

## Results

### Marker-trait associations

**Fig. 1:**
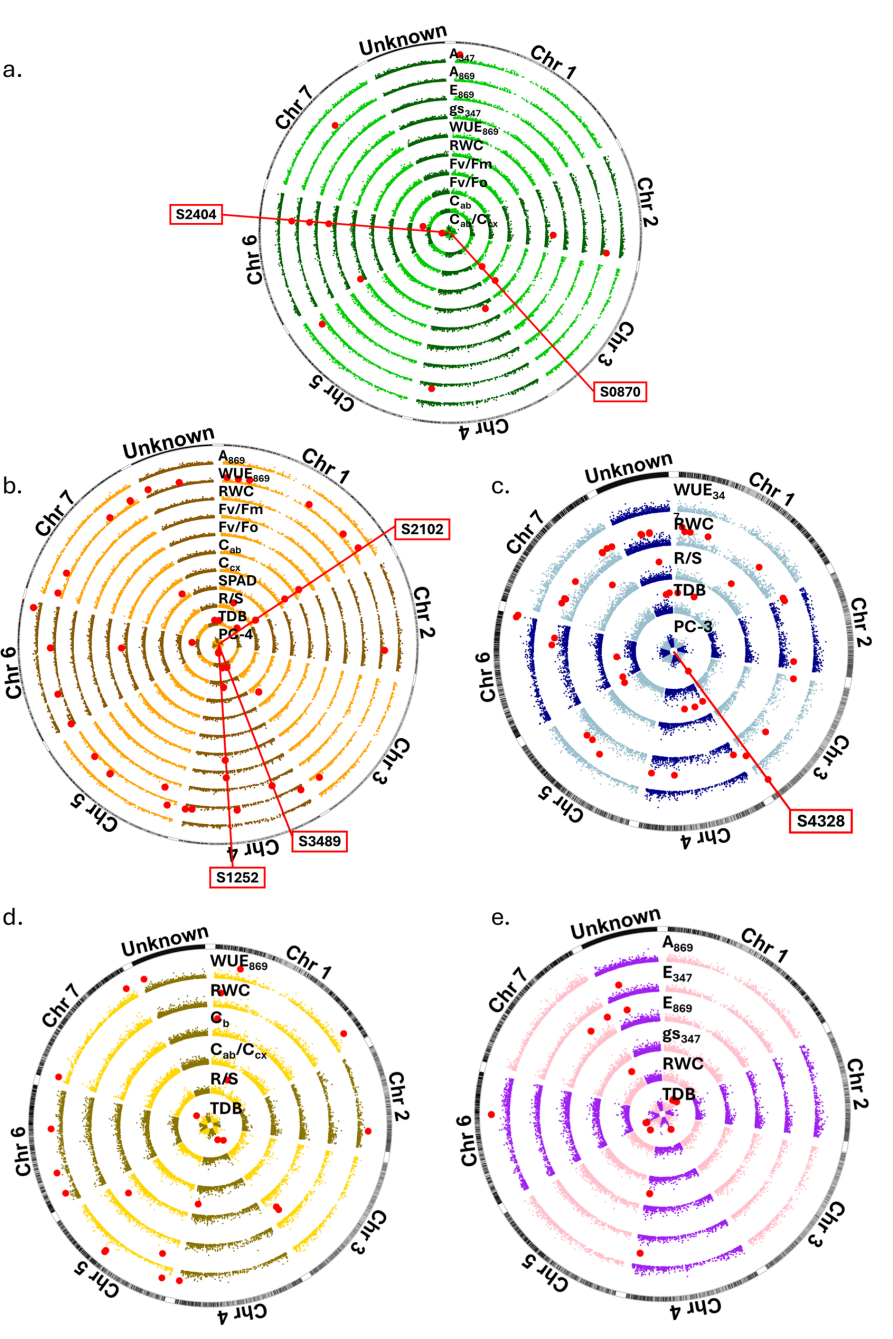
Circular Manhattan plots representing the significant marker-trait associations found in the genome-wide association studies using 194 grass pea accessions, performed with i) BLUEs and ii) eigen values of each accession retrieved from Sanches et al. [82], integrating all the 20 measured traits, under **a**) WW (green), **b**) WD (orange) and **c**) WL (blue) conditions,and the significant marker-trait associations found in the genome-wide association studies performed with iii) delta values calculated for each phenotypic trait and accession in **d**) ΔWD (yellow) and **e**) ΔWL (purple). Each concentring circle depicts the Manhattan plot of a trait, as labelled (A_347_: CO_2_ assimilation rate at growth light intensity (347 μmol/m^2^/s); A_869_: CO_2_ assimilation rate at Amax light intensity (869 μmol/m^2^/s); E_347_: transpiration rate at growth light intensity; E_869_: transpiration rate at Amax light intensity; gs_347_: stomatal conductance at growth light intensity; WUE_347_: instantaneous water use efficiency at growth light intensity (A/E); WUE_869_: instantaneous water use efficiency at Amax light intensity; RWC: leaf relative water content; Fv/Fm: maximum quantum yield of photossystem II; Fv/Fo: actual quantum yield of photossystem II; C_b_: chlorophyll b content (spectrophotometrically measured); C_ab_: chlorophylls a + b content (spectrophotometrically measured); C_cx_: xantophyll + carotene content (spectrophotometrically measured); C_ab_/C_cx_: greeness ratio based on spectrophotometrically measured pigment contents; SPAD: greeness index given by SPAD-502 instrument; R/S: root to shoot dry biomasses ratio; TDB: total dry biomass; PC-3 and PC-4: eigen values of principal components 3 and 4 obtained for each accession x treatment combination in the multivariate analysis previously performed by Sanches et al. [82]. WW: well-watered conditions (21 days with constant soil moisture of about 95% field capacity); WD: water deficit treatment consisting of 16 days of regular watering followed by 5 days of no watering (corresponding to a final soil water content of about 55% FC); WL: waterlogging treatment consisting of well-watered conditions during the first 7 days, followed by 14 days of partial submergence (water reaching approximately 1 cm height of the stem) until measurement day; ΔWD: difference between the Best Linear Unbiased Estimators (BLUEs) of each accession under WD *vs* WW; ΔWL: difference between the BLUEs of each accession under WL *vs* WW.). The y-axis represents the − log10(adj.p) of 5651 SNP markers, and the x-axis exhibits their chromosome position on the grass pea reference genome [100]. Red dots highlight the significantly associated SNPs (FDR method, α = 0.2). Some SNPs with multiple trait associations are further highlighted with their names in labels in a), b) and c). In the outer band of each circular plot, the mapping of SNPs in each grass pea chromosome (+ ‘Unknown’ position) is depicted by black bands

A total of 114 significant marker-trait associations were detected across all GWAS using BLUEs from each trait-treatment combination: 15 under well-watered (WW), 50 under water deficit (WD), and 49 under waterlogging (WL) conditions (Table 1, Fig. 1a-c). Some SNPs were associated with multiple trait-treatment combinations, resulting in a total of 104 SNPs being significantly associated with at least one combination, of which 94 were uniquely mapped to one of the seven chromosomes of the reference grass pea genome [100]. Circular Manhattan plots (Fig. 1) illustrate the distribution of SNPs along the genome (plus unmapped SNPs), with significant associations highlighted in red. Each concentric circle represents a trait-treatment combination.

**Table 1:** Single nucleotide polymorphism (SNP, Marker) significant associations (adj.p-BH: adjusted p-values < 0.01 with an adaptation of Benjamini–Hochberg method, α = 0.2, k = 3007) across 115 independent GWAS, their position within grass pea chromosomes (chr), allelic variants (Ref.Allele/Var.Allele), frequency and effect of the allelic variant, and the proportion of genotypic variance explained by each SNP-trait association (V_QTL_/V_g_) using 194 grass pea accessions subject to well-watered (WW), water deficit (WD) and waterlogging (WL) treatments

GWAS phenotypic input: BLUEs from all the accessions in each trait and treatment combination											
Marker	Trait	Treat	adj.p-BH^a^	Grass Pea genome location (chr^b^ | position (Mpb))		Ref.Allele^c^	Var.Allele^d^	Frequency^e^		Effect^f^	V_QTL_/V_g_ (%)^g^
S0363	A_347_	WW	3.89E-05	1	22.92	G	A	0.216		0.658	0.06
	√WUE_869_	WD	6.22E-04							0.206	0.05
S0294	A_869_	WW	1.94E-04	7	431.67	T	A	0.093		−1.118	0.04
S0644	A_869_	WW	1.56E-04	2	547.76	T	A	0.110		1.066	0.04
S1100	A_869_	WW	7.78E-05	4	608.98	T	C	0.136		1.016	0.04
S1556	A_869_	WW	1.17E-04	5	645.58	C	T	0.098		1.079	0.04
S2404	A_869_	WW	3.89E-05	6	537.36	C	T	0.068		1.643	0.06
	E_869_	WW	3.89E-05							0.671	0.07
	√gs_347_	WW	3.89E-05							0.060	0.06
	TDB	WD	3.89E-05							−0.064	0.05
S2616	A_869_	WD	3.89E-05	6	653.14	T	A	0.079		−1.420	0.07
S3107	E_869_	WL	7.78E-05	5	7.08	C	T	0.143		−0.710	0.06
S2636	√WUE_347_	WL	3.89E-05	6	669.65	A	G	0.119		0.120	0.06
S2672	√WUE_347_	WL	7.78E-05	6	682.29	C	T	0.128		0.108	0.05
S4328	√WUE_347_	WL	1.17E-04	3	679.93	G	A	0.340		0.078	0.06
S3074	√WUE_869_	WW	3.89E-05	2	447.99	A	G	0.157		−0.118	0.08
S3774	√WUE_869_	WW	4.28E-04	6	24.51	G	A	0.110		−0.088	0.03
S0258	√WUE_869_	WD	5.45E-04	1	149.60	T	G	0.108		0.255	0.04
S0303	√WUE_869_	WD	8.95E-04	1	85.90	G	A	0.051		0.365	0.04
S0511	√WUE_869_	WD	9.72E-04	2	459.29	G	A	0.053		0.335	0.04
S0787	√WUE_869_	WD	7.00E-04	3	664.13	G	A	0.069		0.283	0.03
S1051	√WUE_869_	WD	3.89E-05	1	871.77	A	C	0.096		0.368	0.08
S1389	√WUE_869_	WD	5.83E-04	7	142.65	G	C	0.061		0.349	0.05
S1429	√WUE_869_	WD	6.61E-04	4	17.24	C	T	0.092		0.330	0.06
S1513	√WUE_869_	WD	7.78E-05	Unk	23.00	C	G	0.102		0.326	0.06
S1668	√WUE_869_	WD	1.01E-03	5	538.18	G	A	0.069		0.264	0.03
S1839	√WUE_869_	WD	2.72E-04	5	410.28	G	A	0.089		0.315	0.05
S1840	√WUE_869_	WD	1.17E-04	5	410.28	G	C	0.083		0.349	0.06
S1930	√WUE_869_	WD	1.94E-04	5	48.53	G	A	0.052		0.413	0.05
S2133	√WUE_869_	WD	8.17E-04	6	9.43	C	T	0.063		0.326	0.04
S2269	√WUE_869_	WD	2.33E-04	7	22.70	T	G	0.071		0.339	0.05
S2316	√WUE_869_	WD	4.28E-04	6	449.83	G	A	0.109		0.271	0.05
S2318	√WUE_869_	WD	4.67E-04	6	449.83	A	C	0.108		0.271	0.05
S2319	√WUE_869_	WD	4.67E-04	6	449.83	G	T	0.108		0.271	0.05
S2740	√WUE_869_	WD	3.11E-04	7	684.22	C	T	0.067		0.336	0.05
S2742	√WUE_869_	WD	1.56E-04	7	684.22	C	T	0.084		0.344	0.06
S3020	√WUE_869_	WD	1.09E-03	7	537.70	C	G	0.075		0.273	0.03
S3240	√WUE_869_	WD	7.39E-04	1	508.25	A	G	0.093		0.247	0.03
S3395	√WUE_869_	WD	9.33E-04	4	391.86	T	C	0.080		0.272	0.04
S3657	√WUE_869_	WD	8.56E-04	4	644.24	T	A	0.073		0.284	0.04
S3852	√WUE_869_	WD	7.78E-04	1	760.05	C	A	0.190		0.180	0.03
S5038	√WUE_869_	WD	1.05E-03	Unk	198.50	C	T	0.105		0.227	0.03
S5235	√WUE_869_	WD	3.50E-04	4	681.16	T	C	0.061		0.364	0.05
S5478	√WUE_869_	WD	3.89E-04	6	190.05	T	G	0.158		0.242	0.05
S1323	RWC	WW	3.89E-05	4	97.69	T	C	0.444		0.013	0.07
S3489	RWC	WD	7.78E-05	4	159.16	T	G	0.110		−0.035	0.06
	TDB	WD	1.94E-04							0.046	0.04
	TDB	WL	1.56E-04							0.048	0.04
S5474	RWC	WD	3.89E-05	5	94.45	C	A	0.152		−0.036	0.08
S0310	RWC	WL	9.72E-04	1	101.32	C	T	0.061		0.017	0.04
S0339	RWC	WL	1.01E-03	1	58.40	T	C	0.063		0.018	0.04
S0354	RWC	WL	1.17E-04	1	35.50	C	T	0.073		0.018	0.05
S0546	RWC	WL	8.56E-04	7	307.92	A	C	0.186		−0.018	0.12
S0603	RWC	WL	5.45E-04	2	4.57	G	A	0.139		−0.013	0.05
S0932	RWC	WL	3.11E-04	3	478.79	A	G	0.073		0.020	0.06
S1222	RWC	WL	1.56E-04	4	493.01	C	T	0.119		0.013	0.05
S1452	RWC	WL	1.24E-03	4	12.81	T	C	0.287		−0.010	0.05
S1477	RWC	WL	8.95E-04	2	505.94	T	C	0.087		−0.018	0.06
S1708	RWC	WL	8.17E-04	5	509.01	C	T	0.135		−0.013	0.05
S2026	RWC	WL	2.33E-04	3	20.73	C	T	0.152		−0.017	0.09
S2271	RWC	WL	3.89E-04	6	518.08	C	T	0.093		−0.016	0.05
S2507	RWC	WL	9.33E-04	Unk	229.00	A	G	0.148		−0.017	0.09
S2565	RWC	WL	7.00E-04	Unk	230.00	C	T	0.276		0.011	0.06
S2566	RWC	WL	2.72E-04	7	659.91	A	G	0.386		−0.015	0.14
S2681	RWC	WL	3.89E-05	3	707.38	T	A	0.099		−0.017	0.06
S2718	RWC	WL	6.61E-04	7	697.94	T	C	0.102		−0.013	0.04
S2719	RWC	WL	5.83E-04	7	697.94	A	G	0.101		−0.013	0.04
S2872	RWC	WL	6.22E-04	7	612.47	G	T	0.167		−0.011	0.04
S2874	RWC	WL	3.50E-04	7	612.47	G	A	0.166		−0.012	0.05
S2983	RWC	WL	4.28E-04	2	28.92	G	T	0.061		−0.017	0.04
S2996	RWC	WL	1.05E-03	5	454.11	C	A	0.153		0.012	0.05
S3386	RWC	WL	1.09E-03	1	709.44	A	G	0.089		−0.014	0.04
S3670	RWC	WL	1.21E-03	1	243.07	C	T	0.137		0.011	0.04
S3984	RWC	WL	1.13E-03	6	572.88	A	T	0.199		−0.012	0.06
S4424	RWC	WL	4.67E-04	7	111.95	G	A	0.410		0.014	0.12
S4696	RWC	WL	5.06E-04	Unk	163.50	A	G	0.206		−0.017	0.11
S4766	RWC	WL	7.78E-04	Unk	170.00	A	G	0.193		0.010	0.04
S4883	RWC	WL	1.94E-04	4	665.72	T	C	0.156		−0.016	0.09
S4903	RWC	WL	7.78E-05	5	343.93	A	G	0.127		0.015	0.06
S5567	RWC	WL	7.39E-04	7	152.76	G	A	0.257		−0.022	0.2341
S5574	RWC	WL	1.17E-03	7	152.76	G	A	0.296		−0.019	0.1933
S0870	Fv/Fo	WW	3.89E-05	3	570.74	C	G	0.122		−0.147	0.06
	(Fv/Fm)^2^	WW	3.89E-05							−0.009	0.06
S1252	Fv/Fo	WD	3.89E-05	4	441.37	G	C	0.273		0.129	0.06
	(Fv/Fm)^2^	WD	3.89E-05							0.008	0.06
S2661	C_ab_	WW	3.89E-05	6	680.06	A	T	0.077		3.595	0.06
S2102	C_ab_	WD	7.78E-05	1	877.17	G	A	0.201		2.801	0.06
	√C_cx_	WD	3.89E-05							0.109	0.07
S4194	C_ab_	WD	2.72E-04	6	434.21	T	C	0.303		1.971	0.04
S0225	√C_ab_C_cx_	WW	7.78E-05	6	404.15	C	T	0.104		−0.065	0.05
S0837	SPAD	WD	3.89E-05	3	611.22	T	A	0.117		−1.828	0.06
S2857	SPAD	WD	7.78E-05	7	619.24	A	T	0.098		1.741	0.04
S1286	√R/S	WD	1.56E-04	4	377.21	C	T	0.119		−0.066	0.05
S3553	√R/S	WD	3.89E-05	1	898.67	G	A	0.077		0.085	0.05
S5269	√R/S	WD	1.17E-04	1	307.23	G	A	0.050		−0.112	0.06
S5270	√R/S	WD	7.78E-05	1	307.23	C	T	0.050		−0.113	0.06
S0154	√R/S	WL	1.94E-04	1	627.92	C	T	0.052		0.068	0.04
S2977	√R/S	WL	1.17E-04	7	500.86	A	G	0.082		0.060	0.05
S3623	√R/S	WL	7.78E-05	Unk	266.00	A	T	0.057		0.070	0.04
S0018	TDB	WD	1.17E-04	1	772.04	G	A	0.072		0.059	0.04
S0020	TDB	WD	7.78E-05	1	772.04	A	G	0.072		0.059	0.04
S0021	TDB	WD	1.17E-04	1	772.04	C	T	0.072		0.059	0.04
S1291	TDB	WD	3.50E-04	1	15.13	C	T	0.158		0.034	0.03
S3871	TDB	WD	3.11E-04	4	325.57	C	A	0.137		0.038	0.03
	TDB	WL	2.33E-04							0.043	0.04
S4026	TDB	WD	2.33E-04	Unk	290.00	A	T	0.169		0.043	0.05
S0558	TDB	WL	3.89E-04	1	306.46	C	T	0.064		0.061	0.04
S0809	TDB	WL	4.28E-04	4	10.35	G	A	0.066		0.054	0.03
S1463	TDB	WL	3.50E-04	5	694.02	G	C	0.080		0.052	0.03
S2242	TDB	WL	5.45E-04	6	35.53	T	C	0.063		0.053	0.03
S3619	TDB	WL	2.72E-04	1	37.29	T	C	0.092		0.048	0.03
S4108	TDB	WL	4.67E-04	Unk	294.50	C	A	0.197		0.033	0.03
S4764	TDB	WL	3.11E-04	Unk	349.50	C	G	0.190		0.034	0.03
S5238	TDB	WL	5.06E-04	6	377.96	C	A	0.083		−0.049	0.03
GWAS phenotypic input: delta values obtained from subtracting BLUE_stress_—BLUE_WW_ in each trait											
Marker	Trait	Delta	adj.p-BH^a^	Grass Pea genome location (chr^b^ | position (Mpb))		Ref.Allele^c^	Var.Allele^d^	Frequency^e^		Effect^f^	V_QTL_/V_g_ (%)^g^
S2299	A_869_	ΔWL	3.89E-05	6	467.04	T	A	0.076		−1.838	0.05
S1312	√E347	ΔWL	3.89E-05	4	646.15	C	T	0.099		−0.258	0.07
	√gs347	ΔWL	3.89E-05							−0.078	0.05
S1318	√E347	ΔWL	7.78E-05	4	646.15	T	G	0.097		−0.257	0.07
	√gs347	ΔWL	7.78E-05							−0.078	0.05
S4163	√E347	ΔWL	1.17E-04	Unk	114.00	C	A	0.236		−0.156	0.05
	E869	ΔWL	7.78E-05							−0.598	0.05
S2693	E_869_	ΔWL	3.89E-05	7	714.31	T	C	0.107		0.858	0.06
S3023	E_869_	ΔWL	1.17E-04	7	533.59	T	C	0.461		0.576	0.07
S0258	√WUE_869_	ΔWD	3.50E-04	1	149.60	T	G	0.108		0.259	0.04
S0511	√WUE_869_	ΔWD	5.83E-04	2	459.29	G	A	0.053		0.352	0.04
S1051	√WUE_869_	ΔWD	1.17E-04	1	871.77	A	C	0.096		0.326	0.06
S1513	√WUE_869_	ΔWD	1.56E-04	Unk	23.00	C	G	0.102		0.281	0.04
S1839	√WUE_869_	ΔWD	7.78E-05	5	410.28	G	A	0.089		0.359	0.06
S1840	√WUE_869_	ΔWD	3.89E-05	5	410.28	G	C	0.083		0.396	0.07
S1930	√WUE_869_	ΔWD	2.72E-04	5	48.53	G	A	0.052		0.384	0.05
S2269	√WUE_869_	ΔWD	4.28E-04	7	22.70	T	G	0.071		0.331	0.05
S2316	√WUE_869_	ΔWD	3.89E-04	6	449.83	G	A	0.109		0.268	0.04
S2318	√WUE_869_	ΔWD	4.67E-04	6	449.83	A	C	0.108		0.268	0.04
S2319	√WUE_869_	ΔWD	4.67E-04	6	449.83	G	T	0.108		0.268	0.04
S2369	√WUE_869_	ΔWD	3.11E-04	6	62.24	A	G	0.250		0.199	0.05
S2742	√WUE_869_	ΔWD	1.94E-04	7	684.22	C	T	0.084		0.330	0.05
S5235	√WUE_869_	ΔWD	5.45E-04	4	681.16	T	C	0.061		0.336	0.04
S5478	√WUE_869_	ΔWD	2.33E-04	6	190.05	T	G	0.158		0.249	0.05
S5360	RWC	ΔWD	1.94E-04	1	47.51	A	T	0.084		−0.033	0.05
S5474	RWC	ΔWD	3.89E-05	5	94.45	C	A	0.152		−0.030	0.07
S2878	RWC	ΔWL	3.89E-05	7	611.78	G	A	0.085		0.020	0.06
S1073	√C_ab_C_cx_	ΔWD	3.89E-05	4	640.00	C	T	0.462		−0.060	0.08
S0353	∛Cb	ΔWD	7.78E-05	1	35.07	T	C	0.117		0.101	0.08
S0821	∛Cb	ΔWD	1.56E-04	3	634.87	T	C	0.064		0.108	0.05
S0822	∛Cb	ΔWD	3.89E-05	3	634.87	A	G	0.070		0.116	0.06
S1616	∛Cb	ΔWD	1.17E-04	5	581.27	A	G	0.106		0.096	0.06
S5270	√R/S	ΔWD	7.78E-05	1	307.23	C	T	0.050		−0.131	0.06
S0853	TDB	ΔWD	3.89E-05	3	592.85	A	C	0.074		−0.062	0.06
S1462	TDB	ΔWD	1.17E-04	4	54.10	A	G	0.085		0.052	0.04
S5446	TDB	ΔWD	7.78E-05	7	315.61	C	T	0.097		−0.053	0.05
S0002	TDB	ΔWL	1.94E-04	1	772.72	G	A	0.087		0.057	0.04
S0179	TDB	ΔWL	2.72E-04	1	575.70	C	T	0.054		−0.071	0.04
S0180	TDB	ΔWL	3.89E-04	1	575.70	T	C	0.066		−0.061	0.04
S0676	TDB	ΔWL	2.33E-04	6	17.29	T	C	0.089		−0.054	0.04
S1361	TDB	ΔWL	3.11E-04	4	23.17	A	G	0.070		−0.057	0.04
S1872	TDB	ΔWL	7.78E-05	5	364.10	T	C	0.101		−0.051	0.04
S2166	TDB	ΔWL	3.50E-04	6	6.01	T	C	0.155		0.045	0.05
GWAS phenotypic input: PCA coordinates (first four PC's) from all the accessions and treatments											
Marker	PC	Treat	adj.p-BH^a^	Grass Pea genome location (chr^b^ | position (Mpb))		Ref.Allele^c^	Var.Allele^d^	Frequency^e^		Effect^f^	V_QTL_/V_g_ (%)^g^
S4328	PC-3	WL	3.89E-05	3	679.93	G	A	0.340	0.550		0.08
S1254	PC-4	WD	7.78E-05	4	439.15	G	A	0.090	−0.666		0.05
S1255	PC-4	WD	3.89E-05	4	439.15	T	G	0.083	−0.729		0.06

^a^adj.p-BH, adjusted *p*-values < 0.01 (with an adapted Benjamini–Hochberg method, α = 0.2, k = 3007) across 115 independent GWAS, ^b^Chr., chromosome in the grass pea genome, ^c^reference allele, ^d^variant allele, ^e^frequency and ^f^effect of the allelic variant, ^g^the proportion of genotypic variance explained by each SNP-trait association (V_QTL_/V_g_) using 194 grass pea accessions subject to treatments

In a second approach, GWAS were performed using the phenotypic input of ΔWD and ΔWL values (calculated as BLUE_stress_—BLUE_WW_), accounting for the relative effect of water stress on each trait and accession, with more negative Δ values indicating greater trait sensitivity to stress [82]. This method identified 26 and 14 SNPs associated with ΔWD and ΔWL traits, respectively, including three SNPs simultaneously associated with two traits each (Fig. 1d-e). Of the total 40 SNPs associated with Δ traits, 16 overlapped with those identified in the individual trait-treatment GWAS. Thirty-eight of the Δ-associated SNPs were successfully mapped to the grass pea reference genome (Table 1).

Finally, fifteen GWAS were performed across the three water treatments using eigenvalues from the five first principal components (PCs), which together represented 81.15% of the total phenotypic variance in the previous multivariate analysis [82]. Under WD, two SNPs were associated with PC-4, which captured variability in relative water content (RWC), total dry biomass (TDB), root-to-shoot ratio (R/S), and the greenness ratio (C_ab_/C_cx_) [82]. Under WL, one SNP was associated with PC-3, primarily driven by chlorophyll *a* fluorescence related traits [82] and was commonly associated with water use efficiency at 347 μmol/m^2^/s light intensity (WUE_347_) in WL (Table 1, Fig. 1b-c). All three SNPs had known positions in the grass pea genome. No significant associations were detected for any PCs under WW conditions.

Of the 160 marker-trait associations identified (114 from trait-treatment, 43 from Δtraits, and three from PC-treatment GWAS), 103 showed a positive effect of the variant allele relative to the most frequent allele. In the trait-treatment GWAS, a positive effect of the variant allele indicated an increase in the measured trait value. In the Δtrait GWAS, it corresponded to a less negative (or positive) Δ, reflecting reduced susceptibility to WD or WL. In the multivariate GWAS, positive effects represented increased values in traits with positive loadings within the corresponding PC. For example, PC-3 included positive loadings for carbon assimilation rates at 347 and 869 μmol/m^2^/s light intensities (A_347_, A_869_), stomatal conductance and water use efficiency at 869 μmol/m^2^/s light intensity (gs_869_, WUE_869_), WUE_347_, maximum and actual quantum yields of photosystem II (Fv/Fm, Fv/Fo), performance index expressed on absorption basis (PI_ABS_), C_ab_/C_cx_, R/S, and TDB, whereas PC-4 included positive loadings for transpiration rates at 347 and 869 μmol/m^2^/s light intensities (E_347_, E_869_), stomatal conductance and water use efficiency at 347 μmol/m^2^/s light intensity (gs_347_), gs_869_, Fv/Fm, Fv/Fo, PI_ABS_, chlorophyll b and chlorophylls a and b contents (C_b_, C_ab_), C_ab_C_cx_, and R/S [82].

As shown in Table 1, RWC had the highest number of associated markers (38 in total): one SNP under WW, two under WD, 32 under WL, two in ΔWD and one with ΔWL. One SNP (S5474) was common to both WD and ΔWD, but no SNPs were shared across treatments. It was followed by WUE_869_ with 31 associations: two under WW, 28 under WD (14 of which also associated with ΔWD), and one exclusive to ΔWD. No associations were detected for WUE_869_ under WL or ΔWL. TDB was associated with 28 markers: eight under WD, ten under WL (S3871 and S3489 shared), plus three in ΔWD and seven in ΔWL. No associations were found under WW.

In most cases where a single SNP was associated with multiple trait-treatment combinations, the traits were highly correlated [82], Supplementary Figure S1). For instance, S0870 and S1252 were associated with chlorophyll* a* fluorescence traits Fv/Fm and Fv/Fo under WW and WD, respectively; S2102 with pigment content-related traits C_ab_ and carotenes + xanthophylls content (C_cx_) under WD; and S2404 with A_869_, E_869_ and gs_347_ under WW. S0363 was linked to A_347_ under WW and WUE_869_ under WD; S1312 and S1318 with E_347_ and gs_347_ in ΔWL; and S4163 with E_347_ and E_869_ (all gas-exchange related traits). Notably, S2404 was also associated with TDB under WD, making it the marker with the highest number of associations (four). Additionally, S4328 under WL was associated with both WUE_347_ and PC-3, the PC primarily capturing variation in chlorophyll *a* fluorescence-related traits.

Each SNP-trait association accounted for only a small proportion of the observed genotypic variance. The highest individual contributions were from S5567 and S5574, both associated with RWC under WL, explaining 23.41% and 19.33% of the variance, respectively. Regarding association strength, we have observed that there are five cases where the presence of the alternative allele (i.e. the least frequent in the studied collection) conduced to an estimated increase or decrease of more than 80% of the respective trait value: S2661, S2102 and S4294 positive effects over C_ab_ in WW (3.595) and WD (2.801 and 1.971) respectively; and S2299 and S0837 strong negative effects (−1.838 and −1.828) over A_869_ in ΔWL and SPAD in WD respectively. All marker-trait effects are reported in Table 1.

The 4,871 mapped SNPs were evenly distributed across the seven chromosomes of grass pea. The number of markers per chromosome ranged from 476 to 837 in chromosomes 3 and 7, respectively. Under WW conditions, chromosome 6 had the highest number of unique associated markers (four), whereas under WD conditions, chromosomes 1 and 4 had 14 and 10 markers, respectively and under WL conditions, chromosomes 1 and 7 eight and ten associated markers, respectively. These patterns remained consistent after normalising for SNP density across chromosomes. Associations from GWAS using Δ trait values were more evenly distributed. Five and three ΔWD-associated markers were primarily found on chromosomes 1, 5 and 6, and on chromosomes 3, 4 and 7, respectively. The three ΔWL-associated markers were distributed across chromosomes 1, 4, 6, and 7.

We also analysed the chromosomal distribution of associated SNPs by trait group. For gas-exchange-related traits (i.e. A, E, gs and WUE), most of the associated markers mapped on chromosomes 6 and 7 (12 and eight out of the 47 total associations, respectively). For RWC, 27% of all associated markers (10 out of 37) mapped to chromosome 7. Among the 11 markers associated with pigment-related traits (C_a_, C_b_, C_ab_, C_cx_, C_ab_/C_cx_ and SPAD), the greatest number of associations were found on chromosomes 3 and 6 with three markers each. Interestingly, chromosome 3 exhibited the lowest overall SNP density in the dataset.

### Differences in allelic frequencies of associated SNPs among grass pea ecotypes

To compare the frequency of allelic variants of the marker-traits associations across grass pea ecotypes, we defined favourable allele as one causing increase on the trait value. For the associations involving PC-3 and PC-4 or Δ traits, definition of the ‘favourable allele’ required further thought. For example, in ΔWD, the ‘favourable allele’ would be the one that contributes to a less negative (or more positive) Δ value by reducing the impact of the stress relative to WW condition. In contrast, for PC-3-based associations, the ‘favourable’ allele was considered the one that increases the value of the traits with strong positive loadings in the respective component. However, this same allele could be unfavourable for traits contributing negatively for the PC-3 component.

**Fig. 2:**
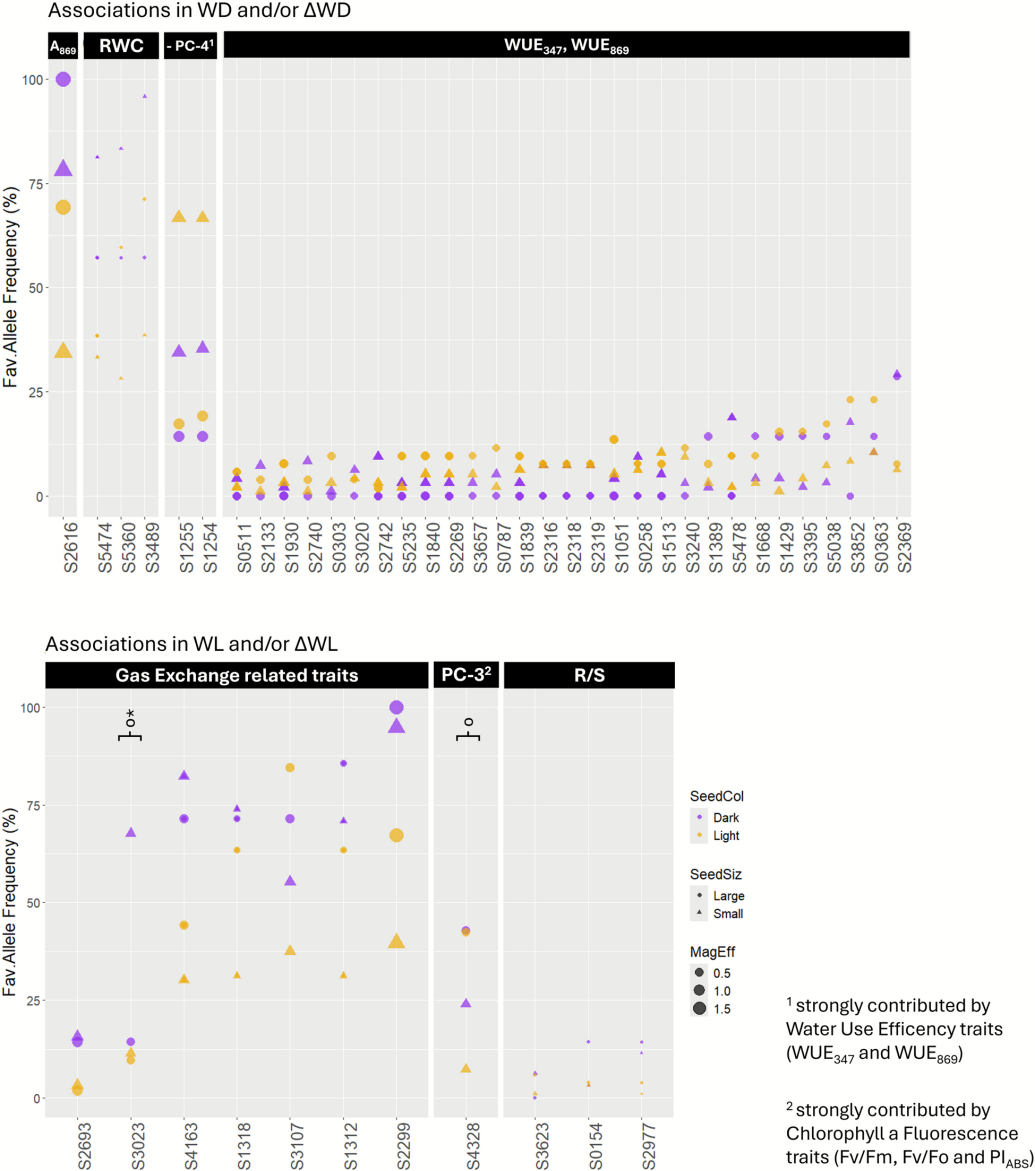
Frequency of the favourable (Fav.) allele in different grass pea seed type clusters, for SNP markers associated with a) A_869_, RWC, PC-4, WUE_347_ and WUE_869_ traits in either WD and/or ΔWD; and b) gas-exchange related traits (specifically gs_347_, E_347_, E_869_ and A_869_), PC-3 and R/S traits (A_869_: CO_2_ assimilation rate at Amax light intensity (869 μmol/m^2^/s); E_347_: transpiration rate at growth light intensity; E_869_: transpiration rate at Amax light intensity; gs_347_: stomatal conductance at growth light intensity; WUE_347_: instantaneous water use efficiency at growth light intensity (A/E); WUE_869_: instantaneous water use efficiency at Amax light intensity; R/S: root to shoot dry biomasses ratio; PC-3: eigen values of principal component 3 obtained for each accession x treatment combination in the multivariate analysis previously performed by Sanches et al. [82]. WD: water deficit treatment consisting of 16 days of regular watering followed by 5 days of no watering (corresponding to a final soil water content of about 55% FC); WL: waterlogging treatment consisting of well-watered conditions during the first 7 days, followed by 14 days of partial submergence (water reaching approximately 1 cm height of the stem) until measurement day; ΔWD: difference between the Best Linear Unbiased Estimators (BLUEs) of each accession under WD *vs* well-watered conditions (WW); ΔWL: difference between the BLUEs of each accession under WL *vs* WW.), in either WL and/or ΔWL, as detected by GWAS using 194 grass pea accessions screened with 5651 SNP markers. Size of dots corresponds to the magnitude of the effect (MagEff) of the favourable allele over the trait, MagEff = 1 meaning that the presence of the favourable allele will increase the trait value with 1 × the average BLUE for that trait (i.e., it will double the average trait value). Shape and colour of dots correspond respectively to seed size and seed colour categories of accessions—purple triangles: dark and small seeded accessions; purple circles: dark and large seeded accessions; yellow triangles: light and small seeded accessions; yellow circles: light and large seeded accessions. Symbols º and * stand for significant differences between favourable allele frequencies (employing Penalized Generalized Linear Mixed Models for binomial data, with α = 0.05 with Bonferroni correction) in seed size and seed colour clusters, respectively

Figure 2 illustrates the traits of greatest physiological relevance under each treatment, focusing on those showing the most pronounced phenotypic differences between seed type clusters [82]. For clarity, seed colour and size were combined to define four accession clusters: dark and small, dark and large, light and small, and light and large seeds.

A comparison of favourable allele frequencies across these grass pea clusters revealed that, in most cases (109 out of 160 associations), seed colour generally had a stronger influence than seed size. However, the effects of seed morphology traits were significant only in a very few cases. Three markers (S1073, S1323, and S2566 associated with C_ab_/C_cx_ in ΔWD, RWC in WW, and RWC in WL, respectively) had significantly higher favourable allele frequency in light, large-seeded accessions (adjusted p-value < 0.05); two markers (S3023 and S4424, associated with E_869_ in ΔWL and RWC in WL, respectively) in dark, small-seeded accessions; and one (S4328, associated with WUE_347_ and PC-3 in WL) in large-seeded accessions. Full details are provided in Supplementary Table S3 and Fig. 2.

For markers S2616, S5474, and S2369, associated respectively with A_869_, RWC and WUE_869_ traits in WD and ΔWD, the favourable allele was present at a higher frequency in dark-seeded accessions. In contrast, for several other markers (S1255, S1254 S1930, S0303, S1840, S2269, S3657, S1839, S2316, S2318, S2319, S1051, S1513, S3240, S3395, S5038, and S0363) associated with WUE traits (WUE_347_, WUE_869_, and the negative PC-4) under the same treatments, the frequency of the favourable allele was higher in light-seeded than in dark-seeded accessions of the same size (Fig. 2a). Regarding seed size, for marker S2616, which has a strong effect on A_869_ under WD conditions, large-seeded accessions had a higher frequency of the favourable allele than small-seeded ones of the same seed colour (Fig. 2a, leftmost panel). A similar pattern was observed for markers S1668, S1429, S3395, S5038, and S0363, all associated with WUE_869_ under WD conditions (with S0363 also linked to A347 under WW conditions). However, for markers S1255 and S1254 (associated with PC-4) and S2742 and S1513 (associated with WUE_869_ under WD and ΔWD), the favourable allele was more prevalent in small-seeded accessions than in large-seeded ones of the same colour.

For almost all markers associated with PC-3, R/S and gas-exchange-related traits under WL and ΔWL (except for S3107 and S3623), the frequency of favourable allele was higher in the dark-seeded accessions than in the light-seeded ones of the same size (Fig. 2b). Regarding seed size, the favourable allele of marker S4163 (linked to E_347_ and E_869_ in ΔWL) was more frequent in small-seeded accessions, whereas marker S4328 (associated with PC-3 in WL and primarily driven by chlorophyll *a* fluorescence traits) exhibited a significantly higher frequency of the favourable allele in large-seeded accessions.

Considering the four groups of accessions defined by seed colour and size, and noting the underrepresentation of ‘light and small’ (yellow triangles) and ‘dark and large’ (purple circles) accessions compared to ‘light and large' (yellow circles) and ‘dark and small' (purple triangles) accessions in the germplasm collection, we established the following. For 22 out of 29 markers associated with WUE in WD/ΔWD (Fig. 2a, right-most panel), and for marker S4329 associated with PC-3 under WL (Fig. 2b, central panel), the light and large-seeded accessions exhibit higher favourable allele frequencies than dark and small-seeded ones. Similarly, across the 28 markers associated with TDB under WD/ΔWD and WL/ΔWL, the favourable allele was more often found at higher frequencies in light and large seeded accessions (Supplementary Table S3). In contrast, for markers associated with A_869_ and RWC under WD/ΔWD (Fig. 2a, leftmost panels), and for most markers associated with gas-exchange traits under WL/ΔWL (six out of seven; Fig. 2b, left panel), the frequencies of the favourable allele was higher in the dark and small-seeded accessions than in the light and large-seeded ones.

**Fig. 3:**
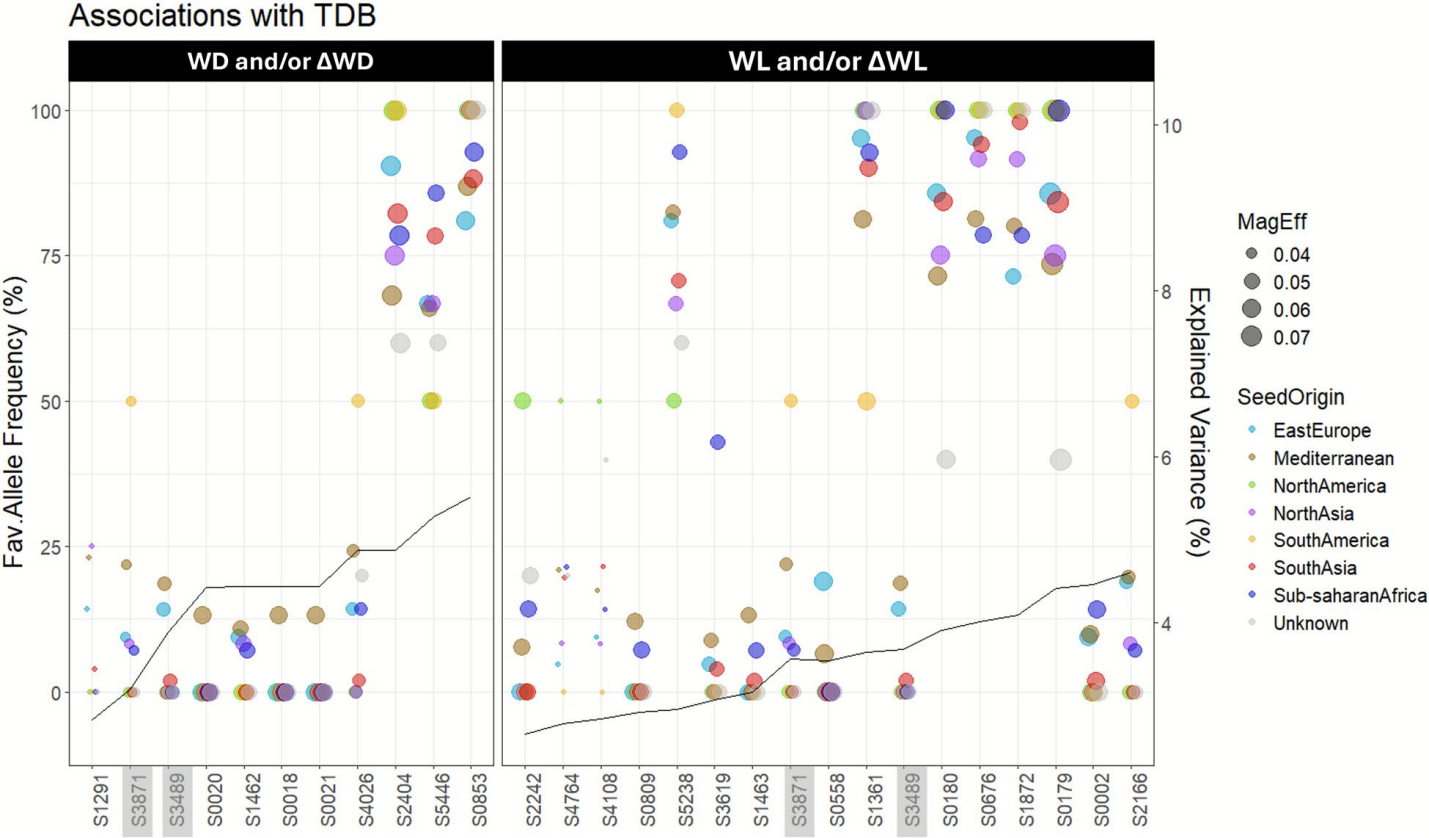
Frequency of the favourable (Fav.) allele in different grass pea geographical origin clusters (eight colours corresponding to seven origins plus one ‘unknown’ category), for markers associated with total TDB in (left panel) WD and/or ΔWD and (right panel) WL and/or ΔWL (WD: water deficit treatment consisting of 16 days of regular watering followed by 5 days of no watering (corresponding to a final soil water content of about 55% FC); WL: waterlogging treatment consisting of well-watered conditions during the first 7 days, followed by 14 days of partial submergence (water reaching approximately 1 cm height of the stem) until measurement day; ΔWD: difference between the Best Linear Unbiased Estimators (BLUEs) of each accession under WD *vs* well-watered conditions (WW); ΔWL: difference between the BLUEs of each accession under WL *vs* WW), as detected by GWAS using 194 accessions screened with 5651 SNP markers. Size of dots corresponds to the magnitude of the effect (MagEff) of the favourable allele over the trait. Markers arranged in each panel in ascending order of the fraction of the trait variance explained by the QTL (a function of total frequency and effect of the favourable allele for each marker), with a black line marking the values (secondary y-axis)

A comparison of allelic frequencies among the geographic origin clusters of grass pea, with a focus on the TDB trait, revealed that markers responsible for a small proportion of the total variance (i.e. those positioned to the left side of the x-axis, Fig. 3), showed the highest favourable allele frequencies in East European and Mediterranean accessions. In contrast, for markers accounting for a large proportion of the variance, South Asian accessions occasionally exhibited a higher favourable allele frequency than the Mediterranean ones. Accessions from Sub-Saharan Africa and North Asia generally displayed frequencies close to the global average, across all markers. Due to the limited number of accessions from South and North America groups (two each), no meaningful patterns could be inferred for these regions.

### Identification of candidate genes and respective functions

To search for putative candidate genes related to WD and WL tolerance, we used the location of the SNPs significantly associated with at least one trait per treatment in the grass pea reference genome by means of Mercator and MapMan analysis for gene identification. Candidate genes were listed only if the SNP mapped within the gene. Of the total of 130 distinct associated markers (namely of the 160 associations, 25, one, and one were common between two, three, and four traits per treatment combinations, respectively), 119 were mapped to a known chromosome position in the grass pea reference genome, of which 41 markers were located in intergenic regions of chromosomes 1 to 7 and several mapped within the same coding DNA sequence, resulting in 66 unique candidate genes.

We examined the functions, gene families, and interaction networks of each candidate gene using the Swiss-Prot and InterPro–UniProt databases and classified them into broad biological functional categories. Candidate genes identified through markers associated with distinct uncorrelated traits (Supplementary Figure S1) and/or stress treatments were prioritized for discussion. Additional genes were also considered where their inclusion was supported by functional interconnectedness and/or recurring biological roles. For the complete list of candidate genes, see Supplementary Table S4.Several of the identified candidate genes seem to be related to carbohydrate metabolism. One example, the chloroplastic starch debranching enzyme isoamylase 1 (Uniprot: O04196), is worth mentioning because three SNPs are located within it and are associated to contrasting water stress conditions, namely S2718 and S2719 (associated with RWC in WL) and S2742 (associated with WUE_869_ in both WD and ΔWD). Another starch-related protein was identified: granule-bound starch synthase 2, chloroplastic/amyloplastic (Uniprot: Q43093), associated with R/S in WL (S0154), whereas S1051, strongly associated with WUE_869_ in both WD and ΔWD, was located within the gene coding for the core subunit of NADH dehydrogenase [ubiquinone] flavoprotein 1, which catalyses the transfer of electrons from NADH through the mitochondrial respiratory chain using ubiquinone as an electron acceptor (UniProt: Q91YT0).

Two candidate genes encoding proteins with redox activity were identified under contrasting water stress conditions: Gfo/Idh/MocA-like oxidoreductase (InterPro: IPR004104), which is related to S3553 associated with R/S in WD, and an aldehyde dehydrogenase family 3 member H1, which is involved in detoxification of lipid peroxidation products (Uniprot: Q70DU8) and related to S1708 associated with RWC in WL. Other genes related to redox signalling mechanisms were found, such as ABC transporter C family member 2. This gene colocalized with S0339 associated with RWC in WL and codes for a pump that transports glutathione S-conjugates (GSH and GSSG, reduced and oxidised forms, respectively).

Candidate genes related to hormonal and transcriptional regulation were also identified. For example, two auxin-responsive genes, auxin response factor 3 (UniProt: O23661) and auxin-responsive protein IAA9 (UniProt: D3K0E6), were associated to RWC in WL (S0354) and R/S in WD (S1286), respectively. S2535 is significantly associated with WUE_869_ in WL and ΔWL and co-localizes with the gene for the PHD finger protein alfin 1 (UniProt: Q9FFF5) gene, a transcription factor involved in plant development and abiotic stress responses.

Finally, some markers linked to the waterlogging context were located in genes involved in cell wall plasticity. For instance, S1312 and S1318, which associated with E_347_ and gs_347_ in ΔWL and S4328, linked to WUE_347_ and PC-3 in WL, localised within two probable pectinesterase/pectinesterase inhibitors (InterPro: IPR000070). Additionally, S0837 with a strong (negative) effect over SPAD trait in WD locates within a gene coding for NETWORKED 1B protein (NET1B, InterPro: F4JIF4), expressed mainly in root meristems with probable developmental- and cytoskeletal-structural related functions. Table 2 lists the abovementioned and other highlighted candidate genes, organised by involvement in similar mechanisms of the water stress response.

**Table 2:** Short list of potentially interesting candidate genes associated with grass pea physiological responses under well-watered (WW), water-deficit (WD), water logging (WL) conditions or with the comparison between water stress and the well water condition (ΔWD and ΔWL co-located with markers associated to at least one trait.treatment found though GWAS using 194 accessions screened with 5651 SNP markers

Marker(s)	Trait(s)	Associated candidate gene and location (bp)^a^	Gene annotation (Unitprot)^b^	Function^c^	Protein involvement	Reason for prioritization
S2718, S2719, S2742	RWC in WL; WUE_869_ in WD and ΔWD	Lsat_g27235 chr7:697,921,143..697952797(bp)	Isoamylase 1, chloroplastic (O04196)	Starch-debranching enzyme involved in amylopectin biosynthesis in endosperm	carbohydrate metabolism	Multiple-trait association
S0154	R/S in WL	Lsat_g2817 chr1:627,912,928..627916929(bp)	Granule-bound starch synthase 2, chloroplastic/amyloplastic (Q43093)	Required for the synthesis of amylose in endosperm	carbohydrate metabolism	Functional interconnectedness and recurring biological roles
S2977	R/S in WL	Lsat_g29796 chr7:500,859,595..500873539(bp)	Fructose-bisphosphate aldolase class-I (P58314)	Essential glycolytic enzyme that catalyses reversible carbon–carbon bond formation	carbohydrate metabolism	Functional interconnectedness and recurring biological roles
S1051	WUE_869_ in WD and ΔWD	Lsat_g855 chr1:871,770,402..871774062(bp)	NADH dehydrogenase [ubiquinone] flavoprotein 2, mitochondrial (Q91YT0)	Catalyzes electron transfer from NADH through the respiratory chain, using ubiquinone as an electron acceptor	carbohydrate metabolism	Functional interconnectedness and recurring biological roles
S0853	TDB in ΔWD	Lsat_g11108 chr3:592,849,973..592854243(bp)	Raffinose synthase or seed imbibition protein Sip1 (Q84VX0)	Involved in the synthesis of raffinose, a major soluble carbohydrate in seeds, roots and tubers	carbohydrate synthesis and osmoprotection	Functional interconnectedness and recurring biological roles
S3553	R/S in WD	Lsat_g517 chr1:898,674,015..898678373(bp)	Gfo/Idh/MocA-like oxidoreductase, C-terminal (InterPro: IPR004104)	Glucose-fructose oxidoreductase?	redox activity and signalling	Recurring biological roles across environments
S1708	RWC in WL	Lsat_g20489 chr5:509,011,641..509014203(bp)	Aldehyde dehydrogenase family 3 member H1 (Q70DU8)	Involved in oxidative stress tolerance by detoxifying reactive aldehydes derived from lipid peroxidation	redox activity and signalling	Recurring biological roles across environments
S0339	RWC in WL	Lsat_g5249 chr1:58,390,157..58407639(bp)	ABC transporter C family member 2 (NCBI GeneID: 125,603,599)	Pump for glutathione S-conjugates such as GSH and GSSG (glutathione oxidized and reduced forms)	redox activity and signalling	Recurring biological roles across environments
S0870	Fv/Fo and Fv/Fm in WW	Lsat_g11301 chr3:570,738,448..570745588(bp)	Calmodulin-interacting protein 111 (Q9LET7)	Enables ATP binding and hydrolysis, locates in cytoplasm and chloroplast	redox activity and signalling	Recurring biological roles across environments
S2102	C_ab_ and C_cx_ in WD	Lsat_g782 chr1:877,166,910. 877,173,278(bp)	ABC transporter Pleiotropic Drug Resistance protein 1 (Q949G3)	Roles on constitutive and jasmonate-driven pathogen defense, by means of antifungal compounds excretion	pathogen defense	Strong effect; multiple-trait association
S0303	WUE_869_ in WD	Lsat_g4983 chr1:85,895,317..85918705(bp)	YT521-B-like domain, 30-kDa cleavage and polyadenylation specificity factor	Probable processing endonuclease. Nucleus-localized, its RNA-binding activity is inhibited by calmodulin in a calcium-dependent fashion	transcriptional/pos-transcriptional regulation	Functional interconnectedness
S5235	WUE_869_ in WD and ΔWD	Lsat_g17909 chr4:681,162,821..681167002(bp)	PHD finger protein Alfin1 (Q9FFF5)	Transcriptional regulator of the salt-inducible PRP2 gene (among others). Plays a role in salinity tolerance in Medicago sativa	transcriptional/pos-transcriptional regulation	Functional interconnectedness
S1361	TDB in ΔWL	Lsat_g14223 chr4:23,170,358..23170802(bp)	FCS-Like Zinc finger 18 (P0DO12)	May act as adapter to facilitate the interaction of SnRK1 complex with effector proteins	transcriptional/pos-transcriptional regulation	Functional interconnectedness
S1286	R/S in WD	Lsat_g15369 chr4:377,207,828..377209628(bp)	Auxin-responsive protein IAA9 (D3K0E6)	Represses early auxin response genes at low auxin concentrations through interaction with auxin response factors	hormonal and transcriptional regulation	Recurring biological roles across environments
S0354	RWC in WL	Lsat_g5491 chr1:35,499,016..35502501(bp)	Auxin response factor 3 (O23661)	Could act as transcriptional activator or repressor. Formation of heterodimers with Aux/IAA proteins may alter their ability to modulate early auxin response genes expression	hormonal and transcriptional regulation	Recurring biological roles across environments
S1312, S1318	E_347_ and gs_347_ in ΔWL	Lsat_g14828 chr4:111,351,305..111354460(bp)	Probable pectinesterase/pectinesterase inhibitor 47 (Q9FF77; InterPro: IPR000070)	Acts in the modification of cell walls via demethylesterification of cell wall pectin	cell structure and dynamics	Functional interconnectedness and recurring biological roles
S4328	WUE_347_ and PC-3 in WL	Lsat_g10076 chr3:679,926,871..679929017(bp)	Probable pectinesterase/pectinesterase inhibitor 41 (Q8RXK7; InterPro: IPR000070)	Acts in the modification of cell walls via demethylesterification of cell wall pectin	cell structure and dynamics	Multiple-trait association; functional interconnectedness and recurring biological roles
S0837	SPAD in WD	Lsat_g10884 chr3:611,217,767 ..611223852(bp)	NETWORKED 1B (F4JIF4)	Expressed mainly in root meristems with probable developmental- and cytoskeletal-structural related functions	cell structure and dynamics	Strong effect (C_ab_); multiple-trait association

^a^From grass pea L007 reference genome [100]

^b^From Mercator4 v2.0 (https://www.plabipd.de/portal/mercator4) or Swissprot (https://www.uniprot.org/)

^c^From Interpro-UniProtKB (https://www.uniprot.org/)

## Discussion

*Lathyrus sativus* is a grain legume that has attracted interest as a promising climate-change proof crop due to its phenotypic and physiological superiority in more marginal environments [5, 30, 43, 55, 98]. However, the genetic and molecular basis of its abiotic stress tolerance has been less studied than that of other grain legumes (Dixit et al. 2016), resulting in limited understanding and hindering efforts in precision breeding for multi-stress resistance.

In this study, a geographically diverse panel of 194 grass pea accessions, comprising broad morphological and genetic variation, enabled a robust GWAS approach to dissect the genetic basis of the species water stress tolerance. In total, 160 significant associations were detected across 20 physiologically relevant traits and five principal components derived from individual water treatments or differentials of stress-control treatment. These results confirmed that tolerance to water deficit and waterlogging in grass pea is a quantitative trait, governed by numerous genes, each contributing modestly to phenotypic variation. Several SNPs were shared among traits, but rarely among treatments, hinting that drought and flood tolerances in grass pea rely on independent genetic architectures, although sometimes related to common molecular processes (namely redox regulation or carbohydrate metabolism), as evidenced by the 66 identified candidate genes. The most strongly associated SNPs with the largest phenotypic effects represent promising targets for the development of molecular tools to support precision breeding for multi-stress tolerance in grass pea and related species.

### The mostly non-concomitant grass pea’s tolerance to WD and WL is achieved by various independent mechanisms

Among the 160 significant SNP-trait associations identified, 24 SNPs were associated in more than one GWAS, resulting in a final set of 130 unique associated SNPs (Table 1, Supplementary Table S1). These multiple associations involved either highly correlated traits or related stress conditions. For example, marker S2404 was simultaneously associated with A_869_, E_869_, and gs_347_ under WW conditions (all gas-exchange-related traits), whereas marker S5474 was associated with RWC in both WD and ΔWD.

Only two SNPs (S3489 and S3871) were associated with the same trait, TDB, in both WD and WL and S3489 also with RWC in WD. As TDB exhibited the highest heritability and was minimally affected by water treatment [82], a great overlap of associated markers across conditions was expected. Another case (see below) involves a candidate gene containing three SNPs, associated with traits under contrasting water stresses. The scarcity of such double-stress associations highlights the different genetic architectures underlying the drought and flood tolerances in grass pea, in line with previous phenotypic data [82] that showed that only two accessions exhibited similar physiological response to both stresses (PI283546, double tolerant and PTLS1001, double susceptible to WD and WL). Overall, our research supports the conclusion that drought and flood tolerance of grass pea do not necessarily coincide on the same germplasm. This interpretation is consistent with the literature, in which natural tolerance to both extremes is rare. Nonetheless, once the genetic basis of individual stress tolerance has been clarified, stress tolerances might be combined, as has been demonstrated in rice (*Oryza sativa*), where marker-assisted selection enabled the development of varieties tolerant to salinity, drought, and submergence by pyramiding beneficial alleles from different inbred lines [63].

Although PI283546 had been classified as tolerant to both WD and WL [82], analysis of this accession’s alleles at stress response-associated markers did not reveal the expected clear enrichment of favourable alleles, or a depletion of unfavourable ones (Supplementary Table S3). This finding may be due in part to the low frequency of alternative alleles at almost all the screened markers (the presence of numerous ‘rare’ alleles makes comparisons of favourable allele counts across accessions difficult). Additionally, the absence of obvious ‘patterns’ of favourable/unfavourable alleles suggest that grass pea tolerance to WD and WL may arise from various different mechanisms, operating independently or in parallel.

Most SNP-trait associations were identified under water stress conditions, with 50 in WD, 49 in WL, and only 15 in WW. Such complexity was anticipated because abiotic stress tolerance typically involves multiple pathways and intricate gene interactions (for a review, see [47] and specifically for grain legumes, [5]), and is generally more complex at the molecular and genetic levels than biotic stress resistance [94, 97]. For instance, we detected many more marker-trait associations than in previous GWAS studies conducted with the same grass pea collection, which focused on biotic stresses [58, 59, 80].

Consistent with the polygenic nature of abiotic stress tolerance, each of the 160 significant SNP-trait associations explained only a small proportion of the phenotypic variance, ranging from 2.67% (S2242 associated with TDB in WL) to 23.41% (S5567 associated with RWC in WL), with a mean of 5.42% (Table 1). Consequently, validating the detected associations is complicated and genomic regions with an increased association density, such as chromosome 7, should be prioritized for QTL inspection [106, 107].

### Identification of preferential genetic targets for assisted breeding, despite the genetic complexity of grass pea water stress tolerance

Stomatal closure is one of the earliest plant responses to water shortage [104], quickly impairing photosynthesis [12, 23]. The 29 SNP markers significantly associated with WUE_869_ (calculated as the ratio of carbon assimilation and transpiration) in WD or ΔWD (Table 1) demonstrate the physiological complexity of the trait and the central role of gas exchange in regulating available water. Gas exchange and photosynthetic efficiency traits are widely used to assess drought responses and have been central of numerous QTL mapping studies aimed at identifying genetic determinants of stress tolerance [21, 26, 96, 106]. Marker S0363 is particularly interesting because it was associated both with WUE_869_ under WD and A_347_ under WW. This dual association suggests its potential utility in identifying accessions that exhibit drought tolerance and high photosynthetic performance under non-stress conditions concomitantly.

Root-related traits are also frequently evaluated in abiotic stress tolerance studies due to the crucial positive impact of a deep, dense root system in the crops’ capacity to avoid drought under low-moisture conditions [36, 54, 77]. Similarly, leaf pigment contents and chlorophyll *a* fluorescence parameters are widely used to assess the physiological performance of other grain legumes under drought and combined stresses [31, 62, 83, 93]. Markers S5474, S1252, S2102, and S5270 were each associated with multiple traits under WD and/or ΔWD (namely RWC, chlorophyll *a* fluorescence, pigment content, and R/S) and explained 6–8% of phenotypic variance. Given their simultaneous involvement in distinct traits, these markers represent promising targets for use in marker-assisted selection of drought-tolerant accessions.

Furthermore, S2102 was also among the markers with the strongest effect on the C_ab_ trait (effect size = 2.801; Table 1). This suggests that selecting accessions carrying the alternative allele at this locus, particularly when combined with the alternative allele at marker S4194 (which also shows a large effect of 1.971 on the same trait; Table 1), has the potential to result in more than a threefold increase in chlorophyll content under mild drought conditions. Whether such an effect is desirable, however, depends on the breeder’s objectives. An additional interesting feature of S2102 marker is that it locates within *Lsat_g782* gene, encoding the ABC transporter Pleiotropic Drug Resistance protein 1 (PDR1), known to play a major role in pathogen defence in *Nicotiana plumbaginifolia* and other plant species [91]. This makes the locus potentially attractive for breeding aimed at improving combined biotic and abiotic stress tolerance.

Under waterlogging conditions, pigment biosynthesis can be impaired, as observed in tomato (*Solanum lycopersicum*) [74] and mung bean [44], possibly because of deficient nutrient absorption [55]. Most crops also exhibit morphological changes in roots and shoots in response to waterlogging [49]. Consequently, traits such as pigment content, greenness index, shoot growth, and elongation rate are commonly employed in association or linkage studies targeting waterlogging tolerance [9, 45, 57, 88]. Markers S0154, S2977, and S3623 were all significantly associated with R/S under WL, each explaining between 3.8% and 4.6% of the phenotypic variance. Therefore, they represent potential candidates for further validation to differentiate grass pea accessions with distinct responses to WL. However, caution is required when interpreting the biological relevance of ‘favourable’ and ‘unfavourable’ alleles. A statistical association between the ‘favourable’ allele with an increased R/S does not necessarily indicate enhanced root depth or density; these patterns were actually rarely observed in the previous phenotyping [82]. Instead, an increased R/S ratio under WL may result from reduced shoot elongation, which is not inherently advantageous, or from root thickening, possibly due to aerenchyma formation [110]. Further anatomical studies of grass pea roots under WL conditions are needed to clarify these mechanisms.

The trait with the highest number of distinct SNP associations under WL was RWC, totalling 33. This high number of associations was somehow unexpected, given the relatively low phenotypic variability observed for this trait [82]. Nevertheless, the substantial genotypic variability suggests that different molecular mechanisms may be responsible for maintaining RWC under WL conditions, i.e. accessions may achieve similar RWC values by activating different cellular responses driven by allelic variation at various loci.

A total of 28 associations were found for TDB: eight in WD, three in ΔWD, ten in WL, and seven in ΔWL. Interestingly, markers S3489 and S3871 were associated both to WD and WL, reflecting intrinsic differences in biomass productivity rather than stress-specific responses. Marker S2404 is particularly noteworthy: in addition to its association with TDB under WD, it is also linked with A_869_ (with a relatively strong positive effect of 1.643, Table 1), E_869_ and gs_347_ in WW, explaining between 4.9% and 6.6% of phenotypic variation of these traits, respectively. This dual association suggests that S2404 could be useful in selecting accessions with more efficient gas exchange and photosynthesis under optimal watering conditions.

Pigment content and greenness ratio are two commonly used phenotypic traits in association or linkage studies of waterlogging tolerance [9, 16, 45], but no significant associations with pigment-related traits were identified in WL or ΔWL. Nevertheless, PC-3, which integrates a linear combination of chlorophyll *a* fluorescence-related traits [82], was significantly associated with marker S4328, which was also associated with WUE_347_. Despite these associations, the relatively low proportion of phenotypic variance explained (5.6% for WUE_347_ and 7.8% for PC-3) limits the usefulness of this marker for marker-assisted selection.

### Genetic confirmation of previously reported differences in physiological responses among grass pea ecotypes under contrasting water stress conditions

We detected interesting ecotypic response differences to WW, WD, and WL treatments based on seed colour, size, and geographical origin. For instance, favourable alleles for WUE traits relevant to drought response were generally more prevalent in accessions with light-coloured large seeds across the ΔWD and WD marker-trait associations. This observation is in contrast with previous findings, in which dark-coloured, small-seeded accessions exhibited higher WUE_869_ under WD [82]. Strikingly, the highest frequency of favourable alleles was found in dark and/or dark-coloured small-seeded accessions for markers explaining the greatest phenotypic variance (e.g., S1389, S5478, S1668, S1429, S3395, S5038, S3852, S0363, and S2369), suggesting a more subtle allele-ecotype interaction.

For markers associated with A_869_ and RWC in WD and ΔWD, as well as for most markers associated with gas exchange-related traits in WL and ΔWL, dark and small-seeded accessions exhibited a higher favourable allele frequency than light and large-seeded accessions. This finding is consistent with previous phenotypic observations, which showed that dark and small-seeded accessions had significantly higher RWC under WD than light and large-seeded ones, less negative ΔWD of RWC and gas exchange parameters, and positive ΔWL responses in A, E, and gs [82].

Regarding TDB, the typically light and large-seeded accessions of Mediterranean and East European origin were previously reported to show increased TDB across water treatments [82]. We also observed a tendency of higher favourable allele frequencies for TDB-associated markers in these origin groups, although most evident in markers explaining a smaller proportion of phenotypic variance. Conversely, for markers explaining a larger fraction of the total variance, the typically dark and small-seeded accessions of the South Asian origin group occasionally surpassed the Mediterranean one in favourable allele frequencies. Among the 28 TDB associations identified under WD, ΔWD, WL, and ΔWL, favourable alleles were more frequently found in light and large-seeded accessions, supporting early phenotypic findings [82].

### Functional prediction of candidate genes indicates an array of common and specific pathways

Significant advancements have recently been made in the annotation of the grass pea genome. The published chromosome-scale reference genome [100] was used here to map candidate genes for the 119 selected SNP markers. Whereas most of these candidate genes encode proteins of unknown function, we identified a set of genes with possible involvement in responses to water stress (Table 2).

Among the most interesting and exemplary candidate genes, several were involved in carbohydrate metabolism, such as *Lsat_g27235*, associated to WUE_869_ in WD and ΔWD and to RWC in WL; and *Lsat_29796*, and *Lsat_g2817*, associated to R/S in WL. Additionally, *Lsat_g855*, associated to WUE_869_ in WD and ΔWD, encodes the core subunit of a NADH dehydrogenase in the mitochondrial electron transport chain and is therefore related to carbohydrate anabolism. These associations are consistent with previous findings in mung bean, where carbohydrate metabolism was linked to adventitious root development during transient waterlogging [45]. More generally, carbohydrate metabolism plays a critical role during water stress. During transient waterlogging, the breakdown of sugars helps to sustain energy production, compensating for the shift in aerobic-to-anaerobic metabolism that often occurs due to hypoxia [101]. Under water deficit, degradation of large polysaccharides is a key mechanism for plants in several aspects: the increased soluble sugar concentration in stomatal guard cells drives stomata closure,the soluble sugars in the mesophyll or in the root cells allow for osmotic regulation, helping water retention; and broken-down sugars provide energy and carbon when photosynthesis is potentially limited by water stress [87, 92].

Furthermore, the accumulation of soluble sugars and proline play an important role in osmoprotection and has been positively associated with abiotic stress tolerance in other legume species, such as faba bean [1] and chickpea (*Cicer arietinum*) [6]. We identified the candidate gene, *Lsat_g11108,* which is associated to TDB in ΔWD and coding for a protein involved in the synthesis of raffinose, a major soluble carbohydrate in seeds, roots, and tubers. Mutant maize (*Zea mays*) plants lacking the protein raffinose synthase (RAFS) or seed imbibition protein Sip1 hyperaccumulate galactinol and are more sensitive to drought stress, whereas *ZmRAFS* overexpression in *Arabidopsis thaliana* improved drought tolerance via increased myo-inositol [51]. Myo-inositol is a sugar alcohol and product of galactinol hydrolysis catalysed by RAFS, which can play an effective role in signal transduction, cell turgor maintenance, and regulation of ROS [109], all key mechanisms during drought responses.

This leads us to the discussion of three other genes: *Lsat_g517*, associated with R/S in WD, and *Lsat_g20489* and *Lsat_g5249*, associated with RWC in WL. These genes are probably involved in redox mechanisms, which are crucial for plant responses to abiotic stresses. Under both drought and waterlogging, ROS accumulate due to stomatal closure, impaired gas exchange, and over-reduction of the photosynthetic electron transport chain (ETC). Potential ROS generation [33, 46, 65] also occurs at mitochondrial ETC complex III [69]. If the plant’s antioxidant defences are unable to respond adequately, oxidative damage can occur, including lipid peroxidation, protein and DNA degradation, chlorophyll loss, membrane disruption, and cell death [28, 29, 66].

Increased antioxidant activity has been suggested to be a possible mechanism involved in grass pea’s tolerance to drought [37, 95], waterlogging [110], and other general stresses [10, 76]. A recent GWAS in cowpea identified a gene encoding an oxygenase family protein (*Vigun04g161000*) associated with pod weight under normal and water stress conditions [83]. In the same study, three candidate genes were associated to drought response for being involved in photoprotection (avoidance of excessive ROS production in the chloroplast under conditions where the dark reaction of photosynthesis is impaired or restricted): *Vigun07g133400, Vigun07g132700,* and *Vigun07g258000.* Specifically, the first gene encodes a protein with organic solute transporter-related functions; the second encodes a germin-like protein (GLS2-1) with superoxide dismutase activity; and the third encodes a UDP-glycosyltransferase 84a1-related protein (UGT84A1), implicated in the glycosylation of various plant metabolites, such as flavonoids and other antioxidants, thus playing a role in mitigating oxidative stress [17]. Together, these findings reinforce the pivotal role of redox homeostasis and antioxidant defence in conferring resilience to water stress in grass pea and other related legumes.

The oxidative burst triggered at the onset of a stress is not only damaging but might also play an important signalling role [13, 70]. One interesting mechanism is the so-called ROS-wave, in which a Ca^2+^-dependent activation of membrane NADPH oxidases under low O_2_ leads to transient H_2_O_2_ accumulation in adjacent cells and facilitates a rapid, ‘overall’ response [60, 61]. The identified *Lsat_g11301* gene, associated with chlorophyll *a* fluorescence traits in WW, encodes a calmodulin-interacting protein and may therefore be involved in this ROS- and Ca^2+^-mediated signalling.

Another candidate gene involved in transcriptional or post-transcriptional regulation is *Lsat_g4983,* which is associated with WUE_869_ in WD and encodes a YT521-B-like domain from a probable processing endonuclease 30-kDa subunit, binding N6-methyladenosine-modified RNA. A similar YT521-B homology domain-containing protein was detected in association with drought in common bean, as well as a zinc finger protein encoded by the *Phvul.010G032700* gene that was implicated in ABA-mediated drought tolerance [50]. Likewise in alfalfa (*Medicago sativa*), the zinc finger protein gene *MtPHD6* [72] and six others in cowpea [106] were associated with drought-responsive QTLs. Zinc finger proteins, particularly those of the plant homeodomain (PHD) class, are widespread in eukaryotic genomes and frequently involved in abiotic stress responses [71]. We identified two such proteins: the PHD finger protein Alfin1 encoded by *Lsat_g17909,* was associated to WUE_869_ in WD and ΔWD and is known to regulate *MsPRP2* expression in alfalfa roots and to be involved in salt stress tolerance [11], hinting at a broader role in abiotic stress tolerance.

The second zinc finger protein found is FCS-Like Zinc finger 18, which is encoded by *Lsat_g14223* and associated to TDB in ΔWL. This protein is thought to function as an adapter, facilitating the interaction between the SnRK1 complex and effector proteins under heat stress [53]. *SnRK1* genes, particularly *SnRK1.1* and *SnRK1.2*, are central regulators of plant hypoxia responses [7, 52]. Consequently, a QTL associated with germination under flooding was found linked with the SnRK1.1-homologous gene *Phvul.008G039400* in common bean [90]. Flooding is also known to induce *SnRK1* expression in an ABA-dependent manner [7, 52], and the interaction between ABA and SnRK1 has been shown to regulate the miR156/SPL module, mediating adaptive responses to flooding in alfalfa [27].In both WD and WL we characterised marker associations linked to candidate genes encoding auxin-regulated transcription factors: genes *Lsat_g15369* (associated with R/S) and *Lsat_g5491* (associated with RWC), respectively. Auxin signalling is well known to regulate root growth and architecture, both of which are key traits in both drought and waterlogging tolerance in legumes (reviewed by Kathun et al. 2021 and [108], respectively).

Additionally, two candidate genes (*Lsat_g10076* and *Lsat_g14828*) encoding pectinesterase/pectinesterase-inhibitor proteins were associated with several WL tolerance-related traits, including WUE, PC-3, E, and gs. These associations imply a possible role for cell wall plasticity in the response to WL, potentially linked with the shoot elongation commonly observed in this grass pea collection [82], and the known partial submergence escape strategy [8]. Moreover, cell wall degradation is a critical process during ethylene-mediated aerenchyma formation, another flood-adaptive trait [85]. Two cellulose synthase and two pectinesterase inhibitor contigs have previously been reported to be downregulated in rust-susceptible grass pea plants upon fungal inoculation [3], highlighting a broader role in cell wall remodelling in grass pea responses to both abiotic and biotic stresses. At the marker loci within the *Lsat_g10076* and *Lsat_g14828* (the two pectinesterase/pectinesterase-inhibitor genes found in our study), S1312, S1318, and S4328, two grass pea rust-susceptible accessions (NC024582 and NC050432) carried all favourable alleles for E_347_ and gs_347_ under ΔWL, and WUE_347_ and PC-3 under WL. Interestingly, one of these accessions (NC024582) was also previously identified as susceptible under WD [82], suggesting possible links between biotic and abiotic stress susceptibility.

Another protein with a putative structural role was identified associated with SPAD in WD: NET1B, encoded by *Lsat_g10884*. This association had one of the strongest marker–trait effects in our study (S0837 effect = −1.828, Table 1). NET1B belongs to a superfamily of actin-binding proteins and, in *Arabidopsis*, is primarily expressed in the root meristem and early elongation zones, functioning as a membrane–cytoskeleton adaptor. A *net1a net1b* double knockout in Arabidopsis shows a long-root phenotype, suggesting that NET1 proteins contribute to root growth control, likely via effects on plasmodesmatal transport, though the transported signals have not been identified [22]. The majority of the identified candidate genes either encode uncharacterised proteins or contain broadly defined annotation domains, limiting the ability to infer specific roles in responses to water stress. Importantly, novel genes and proteins potentially involved in drought and flood tolerance in grass pea need to be functionally characterised. Overall, the proposed candidate genes should be viewed as hypotheses that help guide future investigations by highlighting recurring functional roles and interconnected pathways likely involved in water-stress responses in grass pea and possibly other grain legumes. Given the highly polygenic nature of abiotic stress tolerance and the large number of associated genomic regions identified, any attempt to pinpoint specific causal genes for drought or waterlogging tolerance in grass pea cannot be robustly supported at this stage.

## Conclusions

This study provides the initial clarification of the genetic basis underlying contrasting water stress tolerance (in terms of drought and waterlogging) in grass pea, making use of the recently published reference genome [100]. A total of 160 significant marker-trait associations were identified across 115 independent GWAS analyses (one per trait-treatment combination), corresponding to 130 unique associated regions due to common SNPs across traits.

The largely non-overlapping loci associated with drought and waterlogging (WD/ΔWD and WL/ΔWL) tolerance, imply that these stress tolerances in grass pea are governed by a distinct and highly polygenic genetic basis. However, the identified candidate genes span a range of biological pathways, which, in some cases, suggest some shared or at least interconnected mechanisms, such as those interlinking with reactive oxygen species, carbohydrate metabolism, and auxin signalling. Nevertheless, some associated loci encode uncharacterised proteins, indicating that novel genes associated with drought and flood tolerance in grass pea remain to be discovered.

The SNP markers identified here are a valuable resource for developing molecular tools to support precision breeding for multi-stress tolerance in grass pea and related species. Furthermore, this study paves the way for the future functional validation and expression analysis of candidate genes, certainly possible thanks to the recent development of a genetic transformation protocol via hairy root in grass pea [99]. These advances bring us closer to unlocking the genetic mechanisms behind water stress resilience in this underutilised yet promising crop.

## Supplementary Information

Supplementary Material 1.

Supplementary Material 2.

Supplementary Material 3.

Supplementary Material 4.

Supplementary Material 5.

## Data Availability

Data will be made available on request.
