# Supplementary material for: Integrating natural variation through GWAS – genetics of drought and flood tolerance in grass pea reveal independent yet interconnected mechanisms
Source: BMC Plant Biol. 2026 Feb 5;26:442. doi: 10.1186/s12870-026-08229-y (PMC12973615; doi:10.1186/s12870-026-08229-y)
Supplement: Supplementary file 1 — Supplementary Material 1. [file 12870_2026_8229_MOESM1_ESM.pdf]

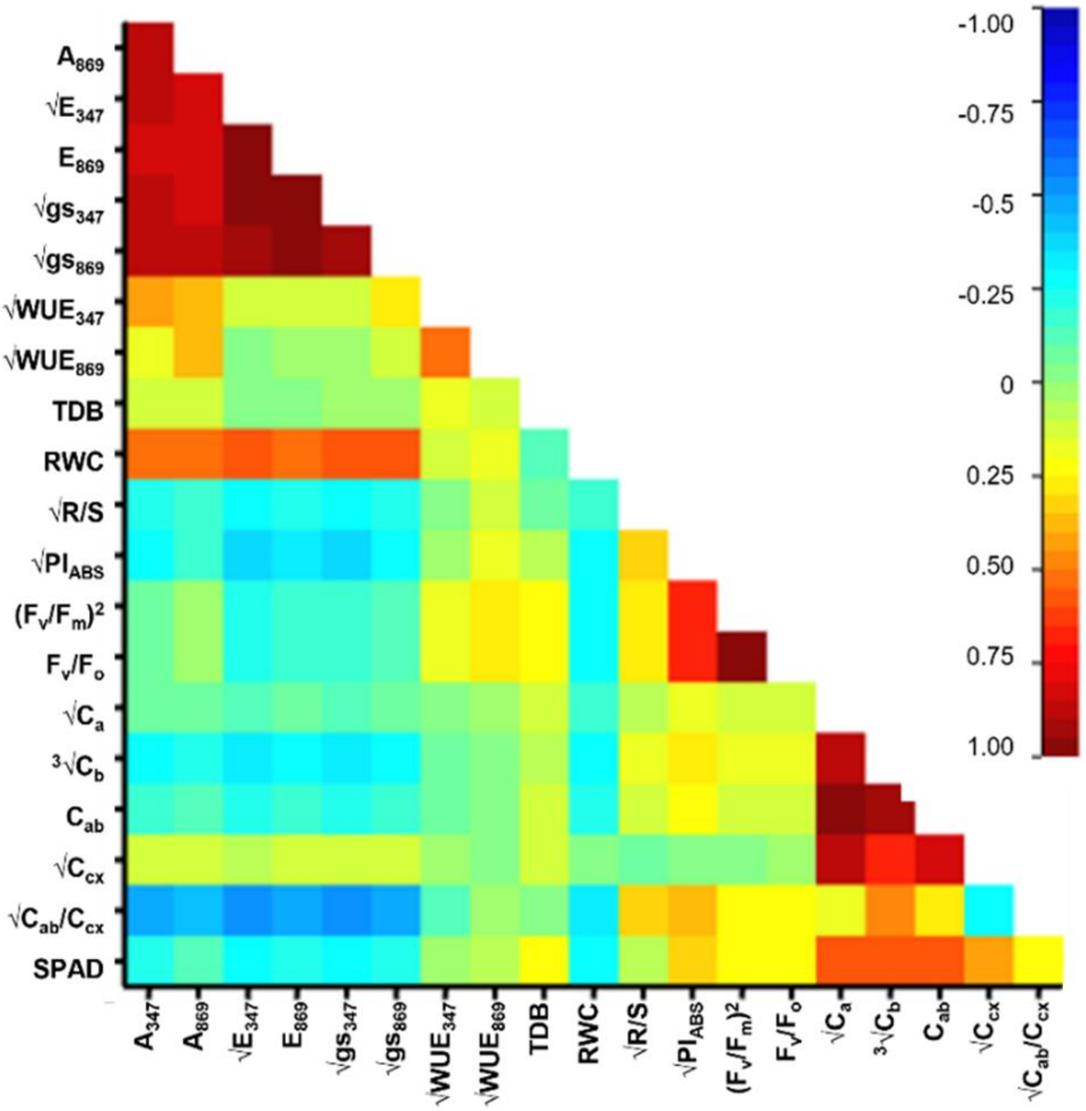

Supplementary Figure S1 – Correlation matrix heatmap with Pearson coefficients based on Best Linear Unbiased Estimators for twenty phenotypic traits measured in 194 grass pea accessions across three distinct water treatments (i.e. Well-watered, Water deficit, and Waterlogging), colored by a spectrum from blue (correlation =−1.0) to red (correlation = 1.0). A_{347}: CO_2 assimilation rate at growth light intensity; A_{869}: CO_2 assimilation rate at Amax light intensity; E_{347}: transpiration rate at growth light intensity; E_{869}: transpiration rate at Amax light intensity; gs_{347}: stomatal conductance at growth light intensity; gs_{869}: stomatal conductance at Amax light intensity; WUE_{347}: instantaneous water use efficiency at growth light intensity; WUE_{869}: instantaneous water use efficiency at Amax light intensity; TDB: total dry biomass; RWC: leaf relative water content; R/S: root to shoot ratio (dry biomasses); PI_{ABS}: performance index on absorbance basis; Fv/Fm: maximum quantum yield of photossystem II; Fv/Fo: actual quantum yield of photossystem II; C_a: chlorophyll a content (spectrophotometrically measured); C_b: chlorophyll b content (spectrophotometrically measured); C_{ab}: chlorophylls a+b content (spectrophotometrically measured); C_{cx}: xantophyll + carotene content (spectrophotometrically measured); C_{ab}/C_{cx}: greeness ratio based on spectrophotometrically measured pigment contents; SPAD: greeness index given by SPAD-502 instrument.
