# Supplementary material for: Integrating natural variation through GWAS – genetics of drought and flood tolerance in grass pea reveal independent yet interconnected mechanisms
Source: BMC Plant Biol. 2026 Feb 5;26:442. doi: 10.1186/s12870-026-08229-y (PMC12973615; doi:10.1186/s12870-026-08229-y)
Supplement: Supplementary file 2 — Supplementary Material 2. [file 12870_2026_8229_MOESM2_ESM.pdf]

Supplementary Table S1 – Seed provider/donor, geographical origins and morphological traits of the 194 grass pea accessions used in the present study. Accession name according to the nomenclature used by the respective plant germplasm bank (PGB), or donor (D). All accessions provided by germplasm banks, as well as accession W6-39226 / Raipur are indexed at Genesys online platform[1].

Source	Accession ID/name	Germplasm Bank Provider or Donor [2]	Seed Origin	Seed Color	Seed Size
PGB	NC006378	CRF-INIA	Mediterranean	Light	Large
PGB	NC006407	CRF-INIA	Mediterranean	Light	Large
PGB	NC006408	CRF-INIA	Mediterranean	Light	Large
PGB	NC011156	CRF-INIA	Mediterranean	Light	Large
PGB	NC012080	CRF-INIA	Mediterranean	Light	Large
PGB	NC012736	CRF-INIA	Mediterranean	Light	Large
PGB	NC013135	CRF-INIA	Mediterranean	Light	Large
PGB	NC013137	CRF-INIA	Mediterranean	Light	Small
PGB	NC020129	CRF-INIA	Mediterranean	Light	Large
PGB	NC020431	CRF-INIA	Mediterranean	Light	Large
PGB	NC024179	CRF-INIA	Mediterranean	Light	Large
PGB	NC024271	CRF-INIA	Mediterranean	Light	Large
PGB	NC024582	CRF-INIA	Mediterranean	Light	Large
PGB	NC035655	CRF-INIA	Mediterranean	Light	Large
PGB	NC035659	CRF-INIA	Mediterranean	Light	Large
PGB	NC038581	CRF-INIA	Mediterranean	Light	Large
PGB	NC038582	CRF-INIA	Mediterranean	Light	Large
PGB	NC047230	CRF-INIA	Mediterranean	Light	Large
PGB	NC047343	CRF-INIA	Mediterranean	Light	Small
PGB	NC049334	CRF-INIA	Mediterranean	Dark	Small
PGB	NC049338	CRF-INIA	Mediterranean	Light	Large
PGB	NC049339	CRF-INIA	Mediterranean	Dark	Small
PGB	NC049346	CRF-INIA	Mediterranean	Dark	Small
PGB	NC050271	CRF-INIA	Mediterranean	Light	Large
PGB	NC050432	CRF-INIA	Mediterranean	Light	Large
PGB	NC050773	CRF-INIA	Mediterranean	Light	Large
PGB	NC050807	CRF-INIA	Mediterranean	Light	Large
PGB	NC050818	CRF-INIA	Mediterranean	Light	Large
PGB	NC069414	CRF-INIA	Mediterranean	Light	Large
PGB	IG64889	ICARDA	Unknown	Dark	Small
PGB	IG64909	ICARDA	Unknown	Dark	Small
PGB	IG64911	ICARDA	Unknown	Dark	Small
PGB	IG64992	ICARDA	Unknown	Dark	Small
PGB	PI163293	USDA	SouthAsia	Dark	Small
PGB	PI165528	USDA	SouthAsia	Light	Small
PGB	PI170469	USDA	Mediterranean	Dark	Small
PGB	PI170470	USDA	Mediterranean	Dark	Small

Source	Accession ID/name	Germplasm Bank Provider or Donor [2]	Seed Origin	Seed Color	Seed Size
PGB	PI172930	USDA	Mediterranean	Dark	Small
PGB	PI174234	USDA	Mediterranean	Dark	Small
PGB	PI179299	USDA	Mediterranean	Dark	Small
PGB	PI179939	USDA	SouthAsia	Dark	Small
PGB	PI180848	USDA	Mediterranean	Dark	Small
PGB	PI183498	USDA	Unknown	Dark	Small
PGB	PI193544	USDA	Sub-saharanAfrica	Dark	Small
PGB	PI193815	USDA	Sub-saharanAfrica	Dark	Small
PGB	PI193994	USDA	Sub-saharanAfrica	Dark	Small
PGB	PI195603	USDA	Sub-saharanAfrica	Dark	Small
PGB	PI195605	USDA	Sub-saharanAfrica	Dark	Small
PGB	PI195993	USDA	Sub-saharanAfrica	Dark	Small
PGB	PI195995	USDA	Sub-saharanAfrica	Dark	Small
PGB	PI195998	USDA	Sub-saharanAfrica	Dark	Small
PGB	PI196001	USDA	Sub-saharanAfrica	Dark	Small
PGB	PI206891	USDA	Mediterranean	Dark	Small
PGB	PI218082	USDA	SouthAsia	Dark	Small
PGB	PI220176	USDA	SouthAsia	Dark	Small
PGB	PI221467_A	USDA	SouthAsia	Dark	Small
PGB	PI221467_B	USDA	SouthAsia	Dark	Small
PGB	PI223269	USDA	SouthAsia	Dark	Small
PGB	PI226948	USDA	Sub-saharanAfrica	Dark	Small
PGB	PI227847	USDA	SouthAsia	Dark	Small
PGB	PI232923_A	USDA	EastEurope	Light	Large
PGB	PI232923_B	USDA	EastEurope	Dark	Large
PGB	PI239866	USDA	SouthAsia	Light	Small
PGB	PI244756	USDA	Sub-saharanAfrica	Dark	Small
PGB	PI251413	USDA	SouthAsia	Dark	Small
PGB	PI255368	USDA	EastEurope	Light	Small
PGB	PI257589	USDA	Sub-saharanAfrica	Dark	Small
PGB	PI268478	USDA	SouthAsia	Dark	Small
PGB	PI269921	USDA	SouthAsia	Dark	Small
PGB	PI283546	USDA	Mediterranean	Dark	Large
PGB	PI283547	USDA	Mediterranean	Dark	Small
PGB	PI283550_A	USDA	NorthAsia	Light	Small
PGB	PI283550_B	USDA	NorthAsia	Dark	Small
PGB	PI283553	USDA	Mediterranean	Light	Small
PGB	PI283554_A	USDA	NorthAsia	Light	Large
PGB	PI283554_B	USDA	NorthAsia	Light	Small
PGB	PI283560	USDA	Mediterranean	Light	Small
PGB	PI283561	USDA	Mediterranean	Light	Small
PGB	PI283562	USDA	SouthAsia	Dark	Small
PGB	PI283564	USDA	Sub-saharanAfrica	Dark	Small

Source	Accession ID/name	Germplasm Bank Provider or Donor [2]	Seed Origin	Seed Color	Seed Size
PGB	PI283565	USDA	Mediterranean	Dark	Small
PGB	PI283566	USDA	Mediterranean	Light	Small
PGB	PI283568	USDA	EastEurope	Light	Small
PGB	PI283569	USDA	Mediterranean	Light	Large
PGB	PI283570_A	USDA	Mediterranean	Light	Small
PGB	PI283570_B	USDA	Mediterranean	Dark	Small
PGB	PI283572	USDA	Mediterranean	Light	Small
PGB	PI283574_A	USDA	Mediterranean	Light	Small
PGB	PI283574_B	USDA	Mediterranean	Dark	Small
PGB	PI283576	USDA	Mediterranean	Light	Small
PGB	PI283578	USDA	Mediterranean	Light	Small
PGB	PI283580	USDA	Mediterranean	Light	Large
PGB	PI283582	USDA	Mediterranean	Dark	Small
PGB	PI283583	USDA	Mediterranean	Dark	Small
PGB	PI283585	USDA	Mediterranean	Dark	Small
PGB	PI283586	USDA	Mediterranean	Dark	Small
PGB	PI283592	USDA	Mediterranean	Light	Small
PGB	PI283593	USDA	EastEurope	Dark	Small
PGB	PI283595	USDA	EastEurope	Light	Small
PGB	PI283597_A	USDA	Mediterranean	Light	Large
PGB	PI283597_B	USDA	Mediterranean	Dark	Small
PGB	PI286531	USDA	SouthAsia	Dark	Small
PGB	PI317439	USDA	SouthAsia	Dark	Small
PGB	PI317443	USDA	SouthAsia	Dark	Small
PGB	PI337087	USDA	SouthAmerica	Light	Small
PGB	PI345525	USDA	SouthAsia	Dark	Small
PGB	PI358600	USDA	Sub-saharanAfrica	Dark	Small
PGB	PI358601	USDA	Sub-saharanAfrica	Dark	Small
PGB	PI358857	USDA	Mediterranean	Dark	Small
PGB	PI380888	USDA	SouthAsia	Dark	Small
PGB	PI383678	USDA	Mediterranean	Light	Small
PGB	PI391430	USDA	SouthAsia	Dark	Small
PGB	PI391431	USDA	SouthAsia	Dark	Small
PGB	PI391432	USDA	SouthAsia	Dark	Small
PGB	PI391433	USDA	SouthAsia	Dark	Small
PGB	PI422521	USDA	EastEurope	Dark	Small
PGB	PI422526_A	USDA	EastEurope	Light	Large
PGB	PI422526_B	USDA	EastEurope	Dark	Large
PGB	PI422527	USDA	EastEurope	Light	Small
PGB	PI422529	USDA	NorthAsia	Light	Small
PGB	PI422532	USDA	NorthAsia	Light	Small
PGB	PI422533	USDA	NorthAsia	Dark	Small
PGB	PI422534	USDA	NorthAsia	Light	Small

Source	Accession ID/name	Germplasm Bank Provider or Donor [2]	Seed Origin	Seed Color	Seed Size
PGB	PI422535	USDA	Mediterranean	Light	Large
PGB	PI422536_A	USDA	Mediterranean	Light	Small
PGB	PI422536_B	USDA	Mediterranean	Dark	Small
PGB	PI422537_A	USDA	EastEurope	Light	Small
PGB	PI422537_B	USDA	EastEurope	Dark	Small
PGB	PI422538	USDA	NorthAsia	Light	Small
PGB	PI422540_B	USDA	Mediterranean	Dark	Small
PGB	PI422541	USDA	NorthAsia	Light	Small
PGB	PI422542	USDA	EastEurope	Light	Small
PGB	PI426880	USDA	SouthAsia	Dark	Small
PGB	PI426882	USDA	SouthAsia	Dark	Small
PGB	PI426884	USDA	SouthAsia	Dark	Small
PGB	PI426886	USDA	SouthAsia	Dark	Large
PGB	PI426888	USDA	SouthAsia	Dark	Small
PGB	PI426890_A	USDA	SouthAsia	Dark	Small
PGB	PI426890_B	USDA	SouthAsia	Light	Small
PGB	PI426892	USDA	SouthAsia	Dark	Small
PGB	PI426894	USDA	SouthAsia	Dark	Small
PGB	PI426896	USDA	SouthAsia	Dark	Large
PGB	PI426897	USDA	SouthAsia	Dark	Small
PGB	PI442793	USDA	SouthAsia	Dark	Small
PGB	PI442795	USDA	SouthAsia	Dark	Small
PGB	PI442797	USDA	SouthAsia	Dark	Small
PGB	PI442799	USDA	SouthAsia	Dark	Small
PGB	PI442801	USDA	SouthAsia	Light	Small
PGB	PI506418	USDA	NorthAmerica	Dark	Small
PGB	PI507931	USDA	EastEurope	Light	Small
PGB	PI511770	USDA	SouthAmerica	Light	Small
PGB	PI513240	USDA	SouthAsia	Dark	Small
PGB	PI513242	USDA	SouthAsia	Dark	Small
PGB	PI513244	USDA	SouthAsia	Dark	Small
PGB	PI513247	USDA	SouthAsia	Dark	Small
PGB	PI513380	USDA	SouthAsia	Dark	Small
PGB	PI543071	USDA	SouthAsia	Light	Small
PGB	PI568190	USDA	Mediterranean	Dark	Small
PGB	PI568191	USDA	Mediterranean	Dark	Small
PGB	PI568195	USDA	Mediterranean	Dark	Small
PGB	PI577138_A	USDA	EastEurope	Dark	Small
PGB	PI577138_B	USDA	EastEurope	Light	Large
PGB	PI577139	USDA	EastEurope	Light	Small
PGB	PI577141	USDA	SouthAsia	Dark	Small
PGB	PI667238	USDA	Mediterranean	Light	Small
PGB	PI667241	USDA	SouthAsia	Light	Small

Source	Accession ID/name	Germplasm Bank Provider or Donor [2]	Seed Origin	Seed Color	Seed Size
PGB	PI667247	USDA	SouthAsia	Dark	Small
PGB	PI667250	USDA	Mediterranean	Light	Large
PGB	PI667251_A	USDA	EastEurope	Light	Large
PGB	PI667251_B	USDA	EastEurope	Dark	Large
PGB	PI667252	USDA	NorthAsia	Dark	Small
PGB	PI667262	USDA	NorthAsia	Dark	Small
PGB	PI667263	USDA	NorthAsia	Light	Small
D	PTLS1001	Maria Emilia Marques	Mediterranean	Light	Large
D	PTLS1002	Vera Lúcia Rodrigues	Mediterranean	Light	Large
D	PTLS1003_A	Irene Alves	Mediterranean	Light	Large
D	PTLS1003_B	Irene Alves	Mediterranean	Light	Large
D	PTLS1004	Nuno Julião	Mediterranean	Light	Large
D	PTLS1005	Maria Isaura Vaz	Mediterranean	Light	Large
D	PTLS1006	Belmira Matos	Mediterranean	Light	Large
D	PTLS1007	Maria Isabel dos Santos	Mediterranean	Light	Large
D	PTLS1008	António José Salvador	Mediterranean	Light	Large
D	PTLS1009	Alexandre Hipólito	Mediterranean	Light	Large
D	PTLS1010	Alice Pisco	Mediterranean	Light	Large
D	PTLS1012	António Costa	Mediterranean	Light	Large
D	PTLS1013	Nuno Julião	Mediterranean	Light	Large
D	PTLS1014	Natália Moniz	Mediterranean	Light	Large
D	PTLS1015	Nuno Almeida	Mediterranean	Light	Large
D	SNVP-64	Susana Neves	Mediterranean	Light	Large
D	Lisa	IAS-CSIC	Mediterranean	Light	Large
D	SITNICA	IFVCNS	EastEurope	Dark	Small
D	STUDENICA	IFVCNS	EastEurope	Light	Large
D	LS87124	Fernand Lambein	NorthAmerica	Light	Small
D	W6-39226 / Raipur	Fernand Lambein	SouthAsia	Dark	Small

[1] https://www.genesys-pgr.org

[2] CRF-INIA: Plant Genetic Resources Centre - National Center of CSIC (Spain); ICARDA: International Center for Agricultural Research in the Dry Areas (Lebanon); USDA: United States Department of Agriculture (USA); IAS-CSIC: Institute of Sustainable Agriculture - Spanish National Research Council (Spain); IFVCNS: Institute of Field and Vegetable Crops, Novi Sad (Serbia).
