# Supplementary material for: Integrating natural variation through GWAS – genetics of drought and flood tolerance in grass pea reveal independent yet interconnected mechanisms
Source: BMC Plant Biol. 2026 Feb 5;26:442. doi: 10.1186/s12870-026-08229-y (PMC12973615; doi:10.1186/s12870-026-08229-y)
Supplement: Supplementary file 3 — Supplementary Material 3. [file 12870_2026_8229_MOESM3_ESM.pdf]

Supplementary Table S2.1 - Description and synthesis of information on the measured traits, average BLUEs values (of all accessions) and variance, per treatment (WW, WD and WL) and per treatment differential (ΔWD and ΔWL); and eigen values of each trait in the first five principal components (PC's) of the multivariate analysis previously elaborated in Sanches et al. (2024). The most influential traits (i.e. with largest eigen values) in each PC are highlighted in blue.

Trait Group	Trait name	Measurement unit	BLUEs Transformation	Trait Acronym	Treatment / Treatment differential (Δ)	Average BLUES	Variance BLUES	Eigen Values				
								PC1	PC2	PC3	PC4	PC5
Gas Exchange **(GasEx)**	CO_2 assimilation rate at growth light intensity (347 μmol/m²/s)	μmol/m²/s	none	**A_{347}**	WW	4.8817	2.2750	-0.324	0.170	0.104	-0.016	-0.022
					WD	2.0962	1.7573					
					WL	6.1484	2.7575					
					ΔWD	-2.7989	3.5056	0.315	0.146	0.126		
					ΔWL	1.2687	4.6075					
	CO_2 assimilation rate at Amax light intensity (869 μmol/m²/s)	μmol/m²/s	none	**A_{869}**	WW	8.7794	5.5270	-0.308	0.197	0.167	-0.016	0.084
					WD	4.7782	4.4378					
					WL	9.7481	5.3365					
					ΔWD	-4.0490	8.3407	0.299	0.185	0.186		
					ΔWL	1.0152	9.4468					
	transpiration rate at growth light intensity (347 μmol/m²/s)	mol/m²/s	square root	**$\sqrt{E_{347}}$**	WW	1.6356	0.0690	-0.347	0.117	-0.028	0.202	-0.004
					WD	0.9307	0.0347					
					WL	2.0291	0.1601					
					ΔWD	-0.7070	0.0972	0.345	0.115	-0.026		
					ΔWL	0.3950	0.1724					
	transpiration rate at Amax light intensity (869 μmol/m²/s)	mol/m²/s	none	**E_{869}**	WW	3.9086	0.8747	-0.337	0.147	-0.007	0.220	0.021
					WD	1.8622	0.4303					
					WL	5.0581	2.2280					
					ΔWD	-2.0609	1.1601	0.333	0.146	0.012		
					ΔWL	1.1537	2.4416					
	stomatal conductance at growth light intensity (347 μmol/m²/s)	mol/m²/s	square root	**$\sqrt{gs_{347}}$**	WW	0.3165	0.0078	-0.346	0.126	-0.003	0.176	-0.046
					WD	0.0817	0.0041					
					WL	0.4453	0.0169					
					ΔWD	-0.2357	0.0113	0.341	0.122	-0.011		
					ΔWL	0.1291	0.0206					
	stomatal conductance at Amax light intensity (869 μmol/m²/s)	mol/m²/s	square root	**$\sqrt{gs_{869}}$**	WW	0.3799	0.0060	-0.339	0.164	0.059	0.129	0.033
					WD	0.1791	0.0068					
					WL	0.4570	0.0127					
					ΔWD	-0.2019	0.0109	0.333	0.157	0.050		
					ΔWL	0.0777	0.0161					
Water Use **(WU)**	instantaneous water use efficiency (A/E) at growth light intensity (347 μmol/m²/s)	ratio	square root	**$\sqrt{WUE_{347}}$**	WW	1.2196	0.0667	-0.087	0.118	0.284	-0.525	0.186
					WD	1.1444	0.2504					
					WL	1.1854	0.0487					
					ΔWD	-0.0734	0.3061	0.069	0.071	0.399		
					ΔWL	-0.0396	0.0937					
	instantaneous water use efficiency (A/E) at Amax light intensity (869 μmol/m²/s)	ratio	square root	**$\sqrt{WUE_{869}}$**	WW	1.4535	0.0457	-0.034	0.110	0.334	-0.456	0.361
					WD	1.4151	0.3107					
					WL	1.3923	0.0281					
					ΔWD	-0.0410	0.3221	0.000	0.084	0.375		
					ΔWL	-0.0640	0.0592					
Leaf water content **(LwC)**	leaf relative water content	%	none	**RWC**	WW	86.5540	0.1125	-0.250	0.014	-0.010	-0.051	0.306
					WD	79.4160	0.4169					
					WL	89.2865	0.0824					
					ΔWD	-7.1460	0.3525	0.254	0.022	-0.004		
					ΔWL	2.7175	0.1050					
	maximum quantum yield of photosystem II	ratio	square	**$(Fv/Fm)^2$**	WW	0.6475	0.0003	0.125	0.151	0.469	0.227	-0.207
					WD	0.6439	0.0004					
					WL	0.6283	0.0004					
					ΔWD	-0.0037	0.0004	-0.163	0.148	0.431		
					ΔWL	-0.0194	0.0005					

Chlorophyll a fluorescence **(ChlF)**	actual quantum yield of photosystem II	ratio	none	**Fv/Fo**	WW	4.1720	0.0772	0.129	0.150	0.466	0.221	-0.212
					WD	4.1273	0.1052					
					WL	3.8804	0.0857					
					ΔWD	-0.0470	0.0995	-0.165	0.144	0.424		
					ΔWL	-0.2941	0.1116					
	Performance index on absorption basis	index	square root	**$\sqrt{PI_{ABS}}$**	WW	2.0278	0.0307	0.191	0.113	0.331	0.242	0.032
					WD	2.0639	0.0498					
					WL	1.7897	0.0320					
					ΔWD	0.0389	0.0519	-0.215	0.093	0.304		
					ΔWL	-0.2377	0.0380					
Pigment content **(PigmC)**	chlorophyll a content (spectrophotometrically measured)	μg/cm²	none	**√Ca**	WW	4.6703	0.1612	0.136	0.435	-0.191	-0.001	0.036
					WD	4.8358	0.2407					
					WL	4.7368	0.1175					
					ΔWD	0.1565	0.2982	-0.114	0.461	-0.170		
					ΔWL	0.0332	0.2273					
	chlorophyll b content (spectrophotometrically measured)	μg/cm²	cubic root	**∛Cb**	WW	1.9994	0.0174	0.205	0.359	-0.163	0.059	0.185
					WD	2.0545	0.0186					
					WL	1.9561	0.0124					
					ΔWD	0.0557	0.0277	-0.194	0.369	-0.201		
					ΔWL	-0.0556	0.0229					
	chlorophylls a+b content (spectrophotometrically measured)	μg/cm²	none	**Cab**	WW	30.2066	29.0301	0.169	0.414	-0.192	0.022	0.080
					WD	32.6435	42.5318					
					WL	30.1067	19.0091					
					ΔWD	2.3670	54.6545	-0.150	0.435	-0.198		
					ΔWL	-0.5776	38.1968					
	xantophyll + carotene content (spectrophotometrically measured)	μg/cm²	square root	**√Ccx**	WW	2.2935	0.0378	0.028	0.427	-0.237	-0.080	-0.099
					WD	2.3494	0.0543					
					WL	2.4365	0.0269					
					ΔWD	0.0487	0.0636	0.006	0.451	-0.174		
					ΔWL	0.1317	0.0511					
Greeness **(Green)**	greeness ratio based on spectrophotometrically measured pigment contents	ratio	square root	**√CabCcx**	WW	2.3725	0.0154	0.234	-0.012	0.073	0.145	0.349
					WD	2.4227	0.0082					
					WL	2.2446	0.0082					
					ΔWD	0.0534	0.0225	-0.230	0.006	-0.008		
					ΔWL	-0.1359	0.0212					
	greeness index given by SPAD-502 instrument	index	none	**SPAD**	WW	34.6124	9.9051	0.169	0.282	-0.014	-0.091	-0.080
					WD	36.7112	12.3538					
					WL	34.4837	12.4980					
					ΔWD	2.1053	11.3769	-0.177	0.226	0.095		
					ΔWL	-0.1399	12.9134					
Dry Biomass **(DryBM)**	root to shoot dry biomasses ratio	ratio	square root	**√R/S**	WW	0.8420	0.0143	0.134	0.013	0.183	0.263	0.438
					WD	0.8430	0.0198					
					WL	0.6930	0.0119					
					ΔWD	0.0010	0.0292	-0.155	0.032	0.048		
					ΔWL	-0.1488	0.0250					
	total dry biomass	g	none	**TDB**	WW	0.3004	0.0201	0.016	0.142	0.149	-0.330	-0.519
					WD	0.2334	0.0106					
					WL	0.2638	0.0124					
					ΔWD	-0.0651	0.0094	0.056	0.119	0.198		
					ΔWL	-0.0329	0.0117					

Supplementary Table S2.2 - Mean and Variance of all coordinates of each accession x treatment combination in the first five principal components (PC's) of the multivariate analysis previously elaborated in Sanches et al. (2024), integrating all the 20 measured traits.

**Trait Group**	**Principal Component (PC)**	**Treatment**	**Mean of PCA coordinates of all accessions**	**Variance of PCA coordinates of all accessions**
GasEx; LwC	**PC1**	WW	-0.5194	1.5181
		WD	2.8758	2.1708
		WL	-2.6716	1.9757
PigmC	**PC2**	WW	0.1400	3.5437
		WD	-0.4710	4.8857
		WL	0.3752	2.8410
ChlF	**PC3**	WW	0.8717	2.0241
		WD	-0.0792	3.0470
		WL	-0.8842	1.7578
WU	**PC4**	WW	0.2722	1.1303
		WD	-0.0785	1.3273
		WL	-0.2156	1.5021
DryBM	**PC5**	WW	0.1541	0.9972
		WD	0.0489	1.6702
		WL	-0.2582	0.6410
