# Supplementary material for: Integrating natural variation through GWAS – genetics of drought and flood tolerance in grass pea reveal independent yet interconnected mechanisms
Source: BMC Plant Biol. 2026 Feb 5;26:442. doi: 10.1186/s12870-026-08229-y (PMC12973615; doi:10.1186/s12870-026-08229-y)
Supplement: Supplementary file 4 — Supplementary Material 4. [file 12870_2026_8229_MOESM4_ESM.pdf]

Supplementary Table S3 - Allelic description and analysis of all the marker-trait associations. Left side of the table: results of the statistical analysis on the favourable allele frequency of different seed colour (light *vs* dark) and seed size (large *vs* small) groups of accessions, using penalized Generalized Linear Mixed Models for binomial data: estimated effects (Est.Effect) of factors 'seed colour' and 'seed size' over the favourable allele frequency of each group; p-values adjusted with Bonferroni correction (adj.p-val, number of tests equals number of markers associated to the same trait and treatment); and specification of which group has a higher favourable allele frequency (> FavAll). For each association, the >FavAll column corresponding to the factor with the larger effect is highlighted. Significant adj.p-val (<0.05) and Markers with which appear repeatedly are also highlighted. Right side of the table: presence of the favourable (fav), unfavourable (unf) or undetermined (-) allele on the 22 previously selected contrasting accessions (Water Deficit (WD) tolerant, Waterlogging (WL) tolerant, WD susceptible and WL susceptible, Sanches et al., 2024). Accessions' origin acronyms: SA-South Asian; NA-NorthAsian; Med-Mediterranean; SS-Sub-saharan Africa; EE-East European; Unk-Unknown. Group of traits' acronyms: GasEx-Gas exchange related traits; WU-Water use; ChlF-Chlorophyll a fluorescence traits; LwC-Leaf water content; PigmC-Pigment contents; Green-Greeness related traits; DryBM-Dry biomass traits. Traits and treatments (Treat) acronyms: as in text.

									Accession	PI317 443	PI577 141	PI391 432	IG649 92	NC03 8582	PI283 546	PTLS 1007	PI358 857	PI196 001	PI422 526B	IG649 09	PTLS 1009	PI667 251A	STUD ENICA	NC02 4582	NC05 0773	PTLS 1001	PI283 578	PI442 795	NC00 6407	NC02 4179	PI422 534
									Seed Colour	Dark	Dark	Dark	Dark	Light	Dark	Light	Dark	Dark	Dark	Dark	Light	Light	Light	Light	Light	Light	Light	Dark	Light	Light	Light
									Seed Size	Small	Small	Small	Small	Large	Large	Large	Small	Small	Large	Small	Large	Large	Large	Large	Large	Large	Small	Small	Large	Large	Small
									Origin	SA	SA	SA	Unk	Med	Med	Med	Med	SS	EE	Unk	Med	EE	EE	Med	Med	Med	Med	SA	Med	Med	NA
				SEED COLOUR			SEED SIZE																								
Group of traits	Trait	Treat	Marker	Est.Effect	adj.p-val	> FavAll	Est.Effect	adj.p-val	> FavAll	Contrastant Group	WD tolerant						WL tolerant					WD susceptible						WL susceptible				
GasEx	A_{347}	WW	S0363	1.110	0.588	Light	0.489	0.806	Large		unf	unf	unf	fav	unf	unf	-	unf	unf	unf	unf	-	-	unf	unf	unf	-	unf	unf	unf	fav	unf
	A_{869}	WW	S0294	0.289	1.000	Dark	0.268	1.000	Small		fav	fav	-	fav	fav	fav	fav	fav	fav	fav	fav	fav	fav	fav	unf	fav	fav	fav	fav	fav	fav	fav
	A_{869}	WW	S0644	0.238	1.000	Dark	0.241	1.000	Large		fav	unf	-	unf	unf	unf	unf	fav	unf	unf	-	unf	unf	unf	unf	unf	unf	unf	unf	unf	unf	unf
	A_{869}	WW	S1100	1.073	1.000	Light	0.151	1.000	Large		unf	unf	-	unf	unf	unf	unf	fav	unf	unf	unf	-	unf	unf	-	-	unf	unf	-	fav	unf	fav
	A_{869}	WW	S1556	1.990	1.000	Light	1.680	1.000	Large		unf	unf	-	unf	unf	unf	unf	unf	unf	fav	unf	fav	fav	unf	unf	unf	fav	unf	unf	unf	fav	unf
	A_{869}	WW	S2404	1.062	1.000	Light	1.167	1.000	Large		unf	unf	-	unf	fav	unf	unf	unf	unf	unf	unf	-	unf	unf	-	unf	-	unf	unf	unf	unf	-
	E_{869}	WW		1.062	0.788	Light	1.167	0.741	Large																							
	gs_{347}	WW		1.062	0.788	Light	1.167	0.741	Large																							
WU	WUE_{869}	WW	S3074	1.888	0.982	Light	2.850	1.000	Large		fav	unf	-	fav	fav	fav	fav	fav	fav	fav	fav	fav	fav	fav	fav	fav	fav	fav	unf	fav	fav	fav
	WUE_{869}	WW	S3774	1.692	1.000	Light	2.416	1.000	Large		-	fav	-	fav	fav	fav	fav	fav	unf	-	fav	fav	fav	fav	fav	fav	fav	fav	-	fav	fav	fav
LwC	RWC	WW	S1323	22.472	1.03E-13	Light	1.108	0.005	Large		-	unf	unf	fav	-	unf	-	unf	unf	unf	unf	-	-	fav	fav	fav	fav	-	-	fav	-	fav
ChlF	Fv/Fm	WW	S0870	2.039	0.565	Light	2.311	0.692	Large		fav	fav	unf	fav	-	fav	fav	-	fav	fav	-	-	fav	fav	fav	fav	fav	fav	fav	fav	fav	fav
	Fv/Fo	WW		2.039	0.565	Light	2.311	0.692	Large																							
PigmC	C_{ab}	WW	S2661	0.675	0.835	Dark	0.556	0.883	Small		unf	unf	-	unf	unf	unf	unf	unf	fav	unf	unf	unf	unf	unf	unf	unf	unf	unf	-	unf	unf	unf
Green	C_{ab}/C_{cx}	WW	S0225	0.414	0.874	Light	0.313	0.916	Large		fav	fav	-	fav	fav	fav	unf	-	unf	fav	fav	fav	fav	fav	fav	fav	fav	unf	fav	fav	fav	fav
GasEx	A_{869}	WD	S2616	1.940	0.662	Dark	1.447	0.660	Small		-	fav	fav	fav	fav	fav	fav	fav	fav	fav	-	-	-	fav	fav	fav	unf	fav	-	fav	fav	-
WU	WUE_{869}	WD	S0258	0.273	1.000	Light	0.567	1.000	Small		unf	-	-	unf	unf	unf	unf	-	unf	unf	fav	unf	unf	fav	unf	unf	unf	fav	unf	unf	fav	unf
	WUE_{869}	Δ WD		0.273	1.000	Light	0.567	1.000	Small																							
	WUE_{869}	WD	S0303	2.297	1.000	Light	1.228	1.000	Large		unf	unf	unf	unf	unf	unf	unf	unf	unf	unf	unf	-	-	fav	unf	-	fav	fav	unf	unf	unf	unf
	WUE_{869}	WD	S0363	1.110	1.000	Light	0.489	1.000	Large		unf	unf	unf	fav	unf	unf	-	unf	unf	unf	unf	-	-	unf	unf	unf	-	unf	unf	unf	fav	unf
	WUE_{869}	WD	S0511	0.348	1.000	Light	0.200	1.000	Large		unf	unf	unf	fav	unf	unf	-	unf	unf	unf	unf	-	-	unf	unf	unf	fav	fav	unf	unf	unf	-
	WUE_{869}	Δ WD		0.348	1.000	Light	0.200	1.000	Large																							
	WUE_{869}	WD	S0787	0.657	1.000	Light	0.817	1.000	Large		unf	unf	-	unf	unf	unf	unf	unf	unf	unf	unf	unf	unf	-	unf	-	unf	unf	unf	unf	unf	unf
	WUE_{869}	WD	S1051	1.403	1.000	Light	0.800	1.000	Large		unf	unf	unf	unf	-	unf	unf	unf	unf	unf	unf	-	-	unf	unf	unf	unf	unf	unf	unf	unf	-
	WUE_{869}	Δ WD		1.403	1.000	Light	0.800	1.000	Large																							
	-PC-4	WD	S1254	0.703	1.000	Dark	0.655	1.000	Small		fav	unf	-	unf	unf	unf	unf	fav	unf	unf	unf	-	unf	unf	unf	-	unf	unf	unf	unf	unf	unf
	-PC-4	WD	S1255	0.933	1.000	Dark	1.085	1.000	Small		fav	unf	-	unf	unf	unf	unf	fav	unf	unf	unf	-	unf	unf	unf	unf	unf	unf	unf	unf	unf	unf
	WUE_{869}	WD	S1389	1.211	1.000	Light	1.109	1.000	Large		unf	unf	unf	unf	-	fav	-	unf	unf	unf	unf	-	-	unf	unf	unf	unf	unf	unf	unf	fav	-
	WUE_{869}	WD	S1429	0.843	1.000	Light	1.766	1.000	Large		-	unf	unf	unf	-	-	-	unf	unf	unf	unf	-	-	unf	unf	unf	unf	unf	-	unf	unf	unf
	WUE_{869}	WD	S1513	1.268	1.000	Light	0.582	1.000	Small		unf	unf	-	unf	unf	unf	unf	unf	-	unf	unf	unf	fav	unf	unf	unf	unf	fav	unf	unf	unf	unf
	WUE_{869}	Δ WD		1.268	1.000	Light	0.582	1.000	Small																							
	WUE_{869}	WD	S1668	0.645	1.000	Light	0.766	1.000	Large		unf	unf	-	unf	unf	unf	unf	unf	unf	fav	unf	unf	fav	unf	unf	unf	unf	unf	unf	fav	unf	-
	WUE_{869}	WD	S1839	1.577	1.000	Light	0.375	1.000	Large		-	-	unf	unf	-	unf	unf	unf	unf	-	unf	-	unf	unf	-	unf	-	unf	unf	unf	-	unf
	WUE_{869}	Δ WD		1.577	1.000	Light	0.375	1.000	Large																							
	WUE_{869}	WD	S1840	1.475	1.000	Light	0.535	1.000	Large		-	-	unf	unf	-	unf	unf	unf	unf	-	unf	-	unf	unf	-	unf	-	unf	unf	unf	-	unf
	WUE_{869}	Δ WD		1.475	1.000	Light	0.535	1.000	Large																							
	WUE_{869}	WD	S1930	1.386	1.000	Light	0.707	1.000	Large		unf	unf	unf	unf	unf	-	unf	-	unf	unf	unf	-	-	unf	unf	-	fav	unf	unf	unf	unf	unf
	WUE_{869}	Δ WD		1.386	1.000	Light	0.707	1.000	Large		unf	unf	unf	unf	unf	-	unf	-	unf	unf	unf	-	-	unf	unf	-	fav	unf	unf	unf	unf	unf
	WUE_{869}	WD	S2133	0.978	1.000	Dark	0.761	1.000	Small		fav	unf	unf	fav	fav	unf	unf	-	unf	unf	unf	-	-	unf	unf	unf	unf	-	unf	unf	unf	unf
	WUE_{869}	WD	S2269	1.563	1.000	Light	0.599	1.000	Large		unf	unf	-	unf	unf	unf	unf	unf	unf	unf	unf	unf	unf	fav	unf	-	unf	fav	unf	-	unf	unf
	WUE_{869}	Δ WD		1.563	1.000	Light	0.599	1.000	Large																							
	WUE_{869}	WD	S2316	0.620	1.000	Light	0.500	1.000	Small		unf	fav	unf	-	unf	unf	unf	-	unf	unf	unf	-	-	unf	unf	unf	unf	unf	unf	unf	unf	unf
	WUE_{869}	Δ WD		0.620	1.000	Light	0.500	1.000	Small																							
	WUE_{869}	WD	S2318	0.647	1.000	Light	0.479	1.000	Small		unf	fav	unf	-	unf	unf	unf	-	unf	unf	unf	-	-	unf	unf	unf	unf	unf	unf	unf	unf	unf
	WUE_{869}	Δ WD		0.647	1.000	Light	0.479	1.000	Small																							
	WUE_{869}	WD	S2319	0.647	1.000	Light	0.479	1.000	Small		unf	fav	unf	-	unf	unf	unf	-	unf	unf	unf	-	-	unf	unf	unf	unf	unf	unf	unf	unf	unf
	WUE_{869}	Δ WD		0.647	1.000	Light	0.479	1.000	Small																							
	WUE_{869}	Δ WD	S2369	1.549	1.000	Dark	1.338	1.000	Small		fav	unf	-	unf	unf	fav	unf	unf	fav	unf	unf	unf	unf	unf	unf	-	unf	-	-	unf	unf	-
	WUE_{869}	WD	S2740	0.979	1.000	Dark	0.902	1.000	Small		unf	fav	unf	unf	unf	unf	unf	unf	-	unf	-	-	fav	unf	unf	unf	unf	unf	unf	unf	unf	unf
	WUE_{869}	WD	S2742	0.891	1.000	Dark	2.019	1.000	Small		unf	fav	unf	unf	unf	unf	unf	unf	-	unf	-	-	-	unf	unf	unf	unf	unf	-	unf	unf	unf
	WUE_{869}	Δ WD		0.891	1.000	Dark	2.019	1.000	Small																							
	WUE_{869}	WD	S3020	0.198	1.000	Light	0.745	1.000	Small		-	-	unf	unf	unf	-	unf	unf	unf	unf	unf	-	-	-	unf	unf	unf	unf	unf	unf	unf	unf
	WUE_{869}	WD	S3240	1.848	1.000	Light	0.167	1.000	Large		unf	unf	-	unf	unf	unf	unf	unf	unf	unf	-	unf	unf	unf	unf	unf	unf	fav	unf	unf	unf	unf
	WUE_{869}	WD	S3395	1.687	1.000	Light	1.513	1.000	Large		unf	unf	-	unf	unf	fav	fav	unf	unf	unf	unf	unf	unf	fav	unf	unf	unf	unf	unf	unf	unf	unf
	WUE_{869}	WD	S3657	1.354	1.000	Light	0.370	1.000	Large		unf	unf	-	unf	fav	unf	unf	unf	unf	unf	unf	unf	unf	unf	unf	unf	unf	unf	unf	unf	unf	fav
	WUE_{869}	WD	S3852	0.300	1.000	Light	0.159	1.000	Large		unf	unf	-	unf	fav	unf	unf	unf	unf	unf	unf	unf	unf	unf	unf	fav	unf	unf	unf	unf	unf	unf
	WUE_{869}	WD	S5038	1.689	1.000	Light	1.071	1.000	Large		unf	unf	-	unf	unf	fav	fav	unf	unf	unf	unf	unf	unf	fav	unf	unf	unf	unf	unf	unf	unf	unf
	WUE_{869}	WD	S5235	0.988	1.000	Light	0.921	1.000	Large		unf	unf	unf	unf	unf	unf	unf	unf	unf	unf	-	-	-	unf	unf	unf	unf	unf	-	unf	unf	unf
	WUE_{869}	Δ WD		0.988	1.000	Light	0.921	1.000	Large																							
	WUE_{869}	WD	S5478	1.169	1.000	Dark	0.663	1.000	Small		fav	fav	unf	unf	-	-	unf	unf	unf	-	unf	-	unf	-	unf	unf	unf	unf	-	unf	unf	unf
	WUE_{869}	Δ WD		1.169	1.000	Dark	0.663	1.000	Small																							
LwC	RWC	WD	S3489	2.138	1.000	Dark	2.439	0.794	Small		fav	fav	-	fav	fav	unf	unf	fav	fav	unf	fav	unf	fav	fav	fav	unf	unf	fav	fav	fav	fav	fav
	RWC	Δ WD	S5360	2.951	1.000	Dark	1.326	1.000	Small		fav	fav	fav	fav	fav	fav	fav	fav	fav	fav	fav	-	-	fav	fav	-	unf	-	fav	unf	fav	fav
	RWC	WD	S5474	2.532	0.917	Dark	2.856	0.543	Small		fav	fav	fav	-	fav	fav	unf	fav	fav	unf	fav	-	-	fav	unf	fav	unf	fav	fav	fav	-	fav
	RWC	Δ WD		2.532	0.917	Dark	2.856	0.543	Small																							
ChlF	Fv/Fm	WD	S1252	0.282	0.865	Light	0.252	0.886	Large		unf	fav	-	unf	unf	unf	unf	fav	fav	unf	-	unf	fav	unf	unf	fav	unf	-	unf	fav	-	fav
	Fv/Fo	WD		0.282	0.865	Light	0.252	0.886	Large																							
PigmC	C_b	Δ WD	S0353	1.445	1.000	Light	0.729	1.000	Large		unf	unf	unf	unf	unf	unf	unf	unf	unf	unf	-	-	fav	unf	-	unf	-	unf	unf	unf	-	-
	C_b	Δ WD	S0821	1.742	1.000	Light	0.854	1.000	Large		unf	unf	unf	unf	unf	unf	fav	unf	unf	unf	unf	-	-	unf	unf	unf	unf	unf	unf	unf	fav	unf
	C_b	Δ WD	S0822	1.841	1.000	Light	1.037	1.000	Large		unf	unf	unf	unf	unf	unf	fav	unf	unf	unf	unf	-	-	unf	unf	unf	unf	unf	unf	unf	fav	unf
	C_b	Δ WD	S1616	1.157	1.000	Light	1.113	1.000	Large		unf	fav	-	unf	-	unf	-	unf	unf	unf	unf	-	-	unf	fav	unf	unf	unf	unf	unf	unf	unf
	C_{ab}	WD	S2102	1.402	1.000	Light	0.720	1.000	Large		unf	unf	unf	unf	unf	unf	unf	unf	fav	unf	-	-	-	unf	unf	fav	fav	unf	unf	unf	fav	-
	C_{cx}	WD		1.402	0.520	Light	0.720	0.721	Large																							
	C_{ab}	WD	S4194	0.228	1.000	Dark	0.440	1.000	Large		unf	unf	-	unf	unf	unf	unf	unf	fav	unf	unf	fav	unf	unf	fav	unf	unf	fav	fav	unf	fav	unf
Green	SPAD	WD	S0837	0.892	1.000	Dark	0.194	1.000	Small		fav	fav	fav	unf	fav	fav	fav	fav	fav	fav	fav	-	fav	unf	fav	fav	fav	fav	fav	-	unf	fav
	C_{ab}/C_{cx}	Δ WD	S1073	1.158	0.001	Light	0.749	0.041	Large		unf	fav	unf	fav	fav	unf	fav	unf	unf	fav	-	-	-	fav	fav	fav	fav	unf	-	unf	fav	-
	SPAD	WD	S2857	0.614	1.000	Light	0.524	1.000	Large		unf	unf	-	unf	unf	-	unf	unf	unf	unf	unf	unf	unf	fav	unf	-	unf	unf	-	-	unf	unf
DryBM	TDB	WD	S0018	19.457	1.000	Light	2.799	1.000	Large		unf	-	unf	unf	unf	unf	fav	unf	unf	unf	unf	-	-	unf	fav	unf	-	unf	unf	-	unf	unf
	TDB	WD	S0020	21.082	1.000	Light	2.808	1.000	Large		unf	-	unf	unf	unf	unf	fav	unf	unf	unf	unf	-	-	unf	fav	unf	-	unf	unf	-	unf	unf
	TDB	WD	S0021	19.457	1.000	Light	2.799	1.000	Large		unf	-	unf	unf	unf	unf	fav	unf	unf	unf	unf	-	-	unf	fav	unf	-	unf	unf	-	unf	unf
	TDB	Δ WD	S0853	1.074	1.000	Dark	1.040	1.000	Small		fav	fav	-	fav	fav	fav	fav	fav	fav	fav	fav	unf	fav	fav	fav	fav	fav	fav	-	unf	fav	fav
	R/S	WD	S1286	0.936	1.000	Dark	0.475	1.000	Small		fav	fav	fav	fav	fav	fav	-	fav	fav	fav	-	-	-	fav	fav	fav	-	fav	-	unf	unf	fav
	TDB	WD	S1291	1.556	1.000	Light	1.242	1.000	Large		unf	-	-	unf	fav	unf	unf	-	unf	unf	unf	-	unf	unf	fav	unf	fav	unf	unf	unf	-	-
	TDB	Δ WD	S1462	1.225	1.000	Light	0.020	1.000	Large		unf	unf	unf	unf	unf	-	unf	-	-	unf	unf	-	unf	unf	fav	unf	unf	-	unf	unf	unf	unf
	TDB	WD	S2404	1.062	1.000	Dark	1.167	1.000	Small		unf	unf	-	unf	fav	unf	unf	unf	unf	unf	unf	-	unf	unf	-	unf	-	unf	unf	unf	unf	-
	TDB	WD	S3489	2.138	1.000	Light	2.439	1.000	Large		fav	fav	-	fav	fav	unf	unf	fav	fav	unf	fav	unf	fav	fav	fav	unf	unf	fav	fav	fav	fav	fav
	R/S	WD	S3553	0.388	1.000	Light	0.304	1.000	Large		unf	unf	-	unf	unf	unf	unf	-	unf	unf	unf	unf	unf	unf	unf	unf	-	unf	unf	unf	unf	-
	TDB	WD	S3871	1.674	1.000	Light	1.820	1.000	Large		unf	unf	-	unf	fav	fav	fav	unf	unf	fav	unf	fav	unf	unf	unf	fav	-	unf	unf	unf	unf	-
	TDB	WD	S4026	1.930	1.000	Light	1.941	1.000	Large		unf	fav	-	fav	fav	fav	fav	unf	unf	unf	unf	fav	fav	unf	fav	unf	unf	unf	unf	unf	fav	unf
	R/S	WD	S5269	0.222	1.000	Light	0.189	1.000	Small		unf	fav	-	fav	fav	fav	fav	fav	fav	fav	fav	-	fav	fav	fav	fav	fav	fav	-	unf	fav	fav
	R/S	WD	S5270	0.250	1.000	Light	0.189	1.000	Small		unf	fav	-	fav	fav	fav	fav	fav	fav	fav	fav	-	fav	fav	fav	fav	fav	fav	-	unf	fav	fav
	R/S	Δ WD		0.250	0.952	Light	0.189	0.965	Small																							
	TDB	Δ WD	S5446	0.416	1.000	Dark	0.479	1.000	Small		-	fav	unf	fav	fav	fav	fav	fav	fav	unf	fav	unf	-	unf	fav	fav	-	-	fav	fav	fav	-

	gs_{347}	Δ WL	S1312	2.121	1.000	Dark	1.701	1.000	Small		fav	fav	fav	fav	fav	fav	fav	fav	fav	fav	fav	-	-	unf	fav	-	fav	unf	-	fav	unf	fav

GasEx	E_{347}	Δ WL	S1312	2.121	1.000	Dark	1.701	1.000	Small	fav	fav	fav	fav	fav	fav	fav	fav	fav	fav	fav			unf	fav		fav	unf		fav	unf	fav
	gs_{347}	Δ WL	S1318	2.149	1.000	Dark	1.760	1.000	Small	fav	fav	fav	fav	fav	fav	fav	fav	fav	fav	fav	-	-	unf	fav	-	fav	unf	-	fav	unf	fav
	E_{347}	Δ WL		2.149	1.000	Dark	1.760	1.000	Small																						
	A_{869}	Δ WL	S2299	2.951	0.622	Dark	2.525	0.498	Small	-	fav	-	fav	fav	fav	fav	fav	fav	fav	fav	fav	fav	unf	-	-	unf	fav	fav	fav	fav	-
	E_{869}	Δ WL	S2693	1.554	1.000	Dark	1.501	1.000	Small	unf	-	-	unf	unf	unf	unf	unf	unf	unf	unf	unf	unf	unf	unf	unf	unf	unf	-	unf	unf	unf
	E_{869}	Δ WL	S3023	22.540	5.83E-15	Dark	2.359	1.42E-05	Small	fav	unf	-	fav	unf	unf	unf	fav	fav	unf	fav	unf	unf	unf	unf	-	unf	unf	-	unf	unf	unf
	E_{869}	WL	S3107	2.286	0.510	Light	1.788	0.669	Large	fav	fav	unf	-	fav	-	fav	fav	unf	fav	unf	-	-	unf	fav	fav	fav	fav	-	fav	-	fav
	E_{347}	Δ WL	S4163	2.107	0.970	Dark	1.860	0.858	Small	fav	fav	-	fav	unf	fav	fav	fav	fav	fav	fav	unf	fav	-	unf	fav	unf	fav	fav	unf	unf	unf
	E_{869}	Δ WL		2.107	0.970	Dark	1.860	0.858	Small																						
WU	WUE_{347}	WL	S2636	1.619	1.000	Light	1.338	1.000	Large	unf	unf	unf	unf	unf	unf	unf	unf	unf	unf	unf	-	-	unf	unf	unf	unf	unf	unf	unf	unf	unf
	WUE_{347}	WL	S2672	2.507	1.000	Light	1.982	1.000	Large	unf	unf	-	unf	unf	unf	unf	unf	-	unf	unf	-	-	unf	unf	fav	unf	unf	unf	unf	-	unf
	WUE_{347}	WL	S4328	0.532	1.000	Light	20.581	9.76E-15	Large	fav	-	-	unf	unf	fav	unf	unf	-	unf	-	fav	fav	unf	fav	unf	unf	unf	unf	unf	unf	unf
LwC	RWC	WL	S0310	0.767	1.000	Light	1.737	1.000	Large	unf	-	-	unf	fav	fav	-	unf	unf	unf	unf	-	unf	unf	-	-	unf	unf	unf	unf	unf	unf
	RWC	WL	S0339	0.492	1.000	Dark	0.635	1.000	Small	unf	unf	-	unf	unf	-	unf	unf	fav	unf	unf	-	-	fav	unf	unf	-	unf	unf	unf	unf	-
	RWC	WL	S0354	0.475	1.000	Dark	0.911	1.000	Small	unf	unf	fav	unf	unf	fav	unf	unf	-	unf	-	-	unf	unf	unf	fav	unf	fav	unf	unf	unf	unf
	RWC	WL	S0546	3.413	1.000	Light	3.108	1.000	Large	unf	-	fav	fav	fav	fav	fav	-	fav	fav	-	-	-	fav	fav	fav	fav	fav	-	fav	fav	fav
	RWC	WL	S0603	1.343	1.000	Dark	1.333	1.000	Small	fav	unf	fav	fav	fav	-	fav	fav	-	fav	fav	-	fav	fav	unf	fav	fav	fav	-	fav	unf	fav
	RWC	WL	S0932	1.364	1.000	Light	1.149	1.000	Large	unf	unf	unf	unf	unf	unf	-	unf	unf	unf	unf	-	-	fav	unf	unf	unf	unf	unf	unf	fav	unf
	RWC	WL	S1222	1.093	1.000	Dark	0.416	1.000	Small	unf	unf	-	unf	unf	unf	unf	unf	unf	unf	fav	unf	fav	unf	fav	unf	unf	unf	unf	unf	unf	unf
	RWC	WL	S1452	2.162	1.000	Light	1.996	1.000	Large	unf	fav	-	fav	fav	fav	fav	fav	fav	fav	-	fav	fav	-	fav	fav	fav	fav	-	fav	fav	-
	RWC	WL	S1477	2.890	1.000	Light	2.014	1.000	Large	fav	-	fav	-	fav	fav	fav	-	fav	fav	fav	-	-	fav	fav	fav	fav	-	-	fav	fav	fav
	RWC	WL	S1708	1.669	1.000	Dark	0.000	1.000	Small	fav	fav	fav	fav	fav	unf	fav	-	fav	fav	fav	-	fav	-	fav	-	fav	-	fav	-	fav	unf
	RWC	WL	S2026	2.123	1.000	Light	1.982	1.000	Large	fav	fav	-	fav	fav	fav	fav	fav	fav	fav	unf	fav	fav	fav	fav	unf	fav	fav	-	fav	fav	fav
	RWC	WL	S2271	1.121	1.000	Dark	0.965	1.000	Small	fav	fav	-	unf	fav	-	unf	fav	fav	fav	fav	fav	fav	fav	fav	unf	fav	fav	fav	unf	fav	fav
	RWC	WL	S2507	2.896	1.000	Light	2.626	1.000	Large	fav	unf	-	fav	fav	fav	-	fav	fav	fav	-	-	fav	fav	fav	fav	fav	fav	-	fav	fav	fav
	RWC	WL	S2565	2.424	1.000	Light	1.207	1.000	Large	unf	unf	-	unf	unf	unf	fav	unf	unf	unf	unf	fav	unf	unf	fav	fav	unf	fav	unf	unf	unf	unf
	RWC	WL	S2566	22.942	4.02E-12	Light	2.679	1.21E-04	Large	unf	unf	-	fav	fav	fav	fav	unf	unf	fav	unf	fav	fav	unf	fav	fav	fav	-	unf	fav	fav	fav
	RWC	WL	S2681	0.160	1.000	Dark	0.537	1.000	Large	fav	fav	-	fav	fav	fav	fav	unf	fav	fav	unf	fav	fav	fav	fav	unf	fav	fav	fav	fav	fav	fav
	RWC	WL	S2718	0.138	1.000	Dark	0.424	1.000	Large	-	fav	fav	fav	fav	fav	fav	-	fav	fav	-	-	unf	unf	fav	fav	fav	fav	fav	fav	fav	-
	RWC	WL	S2719	0.166	1.000	Dark	0.435	1.000	Large	-	fav	fav	fav	fav	fav	fav	-	fav	fav	-	-	unf	unf	fav	fav	fav	fav	fav	fav	fav	-
	RWC	WL	S2872	1.174	1.000	Dark	0.516	1.000	Small	fav	fav	fav	fav	fav	fav	fav	fav	fav	fav	fav	-	-	fav	fav	-	fav	fav	fav	fav	fav	-
	RWC	WL	S2874	1.187	1.000	Dark	0.348	1.000	Small	fav	fav	fav	fav	fav	fav	fav	fav	fav	fav	fav	-	-	fav	fav	-	fav	fav	fav	fav	fav	-
	RWC	Δ WL	S2878	1.140	0.738	Light	1.355	0.663	Large	unf	unf	-	unf	-	unf	fav	unf	unf	unf	unf	-	-	unf	-	unf	unf	unf	unf	unf	fav	unf
	RWC	WL	S2983	1.084	1.000	Dark	0.660	1.000	Small	fav	fav	fav	fav	-	-	fav	fav	fav	fav	fav	-	-	-	fav	fav	fav	-	fav	unf	fav	fav
	RWC	WL	S2996	2.184	1.000	Light	1.767	1.000	Large	unf	unf	unf	unf	fav	unf	fav	unf	unf	unf	unf	-	fav	fav	fav	unf	unf	unf	-	fav	fav	unf
	RWC	WL	S3386	1.013	1.000	Dark	1.091	1.000	Small	fav	fav	-	fav	fav	fav	fav	unf	fav	fav	fav	fav	fav	fav	fav	fav	fav	fav	fav	fav	fav	fav
	RWC	WL	S3670	1.082	1.000	Light	0.932	1.000	Large	unf	unf	-	unf	fav	unf	unf	unf	unf	unf	unf	unf	unf	fav	fav	-	unf	fav	fav	fav	unf	unf
	RWC	WL	S3984	2.154	1.000	Light	2.409	1.000	Large	unf	unf	-	fav	fav	fav	unf	unf	fav	fav	fav	fav	fav	fav	fav	fav	fav	fav	-	fav	unf	fav
	RWC	WL	S4424	22.310	1.83E-14	Dark	2.258	1.66E-04	Small	fav	-	-	unf	unf	unf	-	-	fav	unf	fav	unf	unf	fav	unf	unf	unf	unf	fav	unf	unf	unf
	RWC	WL	S4696	2.429	1.000	Light	2.493	1.000	Large	unf	-	-	fav	fav	fav	fav	fav	fav	fav	fav	fav	fav	fav	fav	fav	fav	fav	unf	fav	fav	fav
	RWC	WL	S4766	2.428	1.000	Light	1.623	1.000	Large	unf	unf	-	unf	unf	unf	unf	unf	unf	unf	unf	fav	fav	fav	-	fav	unf	unf	unf	unf	fav	fav
	RWC	WL	S4883	1.300	1.000	Light	1.074	1.000	Large	fav	fav	-	fav	fav	fav	fav	fav	fav	fav	fav	fav	fav	fav	fav	-	fav	fav	-	fav	fav	fav
	RWC	WL	S4903	1.719	1.000	Light	1.754	1.000	Large	unf	unf	-	unf	fav	unf	unf	unf	unf	unf	unf	unf	unf	fav	unf	unf	unf	unf	unf	unf	unf	unf
	RWC	WL	S5567	2.575	1.000	Light	3.194	1.000	Large	unf	-	fav	fav	fav	-	-	fav	fav	fav	-	-	-	fav	fav	fav	-	fav	-	fav	-	fav
	RWC	WL	S5574	2.668	1.000	Light	2.692	1.000	Large	unf	-	fav	fav	fav	-	-	fav	fav	fav	-	-	-	fav	fav	fav	-	fav	-	fav	-	fav
ChlF	PC-3	WL	S4328	0.532	0.722	Light	20.581	3.25E-15	Large	fav	-	-	unf	unf	fav	unf	unf	-	unf	-	fav	fav	unf	fav	unf	unf	unf	unf	unf	unf	unf
DryBM	TDB	WL	S0002	0.758	1.000	Light	0.299	1.000	Large	unf	-	unf	unf	fav	unf	fav	unf	-	unf	unf	-	-	unf	unf	unf	unf	unf	unf	unf	unf	unf
	R/S	WL	S0154	0.244	1.000	Light	0.188	1.000	Large	unf	unf	unf	unf	unf	-	unf	unf	unf	fav	unf	-	-	unf	unf	unf	unf	unf	unf	unf	-	unf
	TDB	Δ WL	S0179	0.312	1.000	Dark	0.773	1.000	Small	fav	fav	fav	unf	fav	fav	fav	fav	fav	fav	fav	-	-	fav	fav	-	unf	fav	fav	fav	unf	-
	TDB	Δ WL	S0180	0.690	1.000	Dark	1.249	1.000	Small	fav	fav	fav	unf	fav	fav	fav	fav	fav	fav	fav	-	-	fav	fav	-	unf	fav	fav	fav	unf	-
	TDB	WL	S0558	1.742	1.000	Light	2.120	1.000	Large	unf	-	unf	unf	-	unf	unf	unf	unf	unf	unf	-	-	fav	unf	unf	unf	unf	unf	unf	unf	unf
	TDB	Δ WL	S0676	1.402	1.000	Dark	1.183	1.000	Small	fav	fav	-	fav	fav	fav	fav	fav	-	fav	fav	unf	fav	fav	unf	fav	fav	fav	unf	fav	unf	unf
	TDB	WL	S0809	0.820	1.000	Light	0.956	1.000	Large	unf	unf	-	unf	unf	fav	unf	-	unf	unf	unf	unf	unf	unf	fav	unf	unf	unf	-	unf	unf	unf
	TDB	Δ WL	S1361	1.038	1.000	Dark	0.861	1.000	Small	fav	fav	-	fav	fav	fav	fav	fav	fav	fav	fav	fav	fav	fav	fav	fav	fav	unf	-	unf	-	fav
	TDB	WL	S1463	1.113	1.000	Light	2.441	1.000	Large	unf	unf	unf	unf	unf	fav	fav	unf	unf	unf	unf	-	unf	unf	fav	unf	fav	unf	unf	unf	unf	unf
	TDB	Δ WL	S1872	0.987	1.000	Dark	0.886	1.000	Small	fav	fav	-	fav	unf	fav	fav	fav	fav	fav	fav	unf	fav	unf	fav	fav	fav	unf	fav	fav	fav	-
	TDB	Δ WL	S2166	2.127	1.000	Light	1.350	1.000	Large	unf	unf	unf	unf	unf	unf	fav	unf	unf	fav	unf	-	-	unf	unf	-	fav	fav	unf	unf	unf	unf
	TDB	WL	S2242	0.164	1.000	Dark	0.245	1.000	Large	unf	unf	unf	unf	unf	fav	unf	unf	unf	unf	fav	-	-	unf	unf	unf	unf	unf	unf	unf	unf	unf
	R/S	WL	S2977	1.545	1.000	Dark	0.598	1.000	Small	unf	unf	-	-	unf	unf	unf	unf	unf	fav	unf	unf	unf	unf	unf	unf	unf	unf	-	unf	unf	unf
	TDB	WL	S3489	2.138	1.000	Light	2.439	1.000	Large	fav	fav	-	fav	fav	unf	unf	fav	fav	unf	fav	unf	fav	fav	fav	unf	unf	fav	fav	fav	fav	fav
	TDB	WL	S3619	0.124	1.000	Light	0.853	1.000	Large	unf	unf	-	unf	unf	unf	fav	unf	fav	-	unf	fav	fav	unf	unf	unf	unf	unf	unf	unf	unf	unf
	R/S	WL	S3623	0.358	1.000	Dark	0.045	1.000	Large	unf	unf	-	unf	unf	unf	unf	unf	unf	unf	unf	unf	-	unf	unf	unf	unf	unf	unf	-	unf	unf
	TDB	WL	S3871	1.674	1.000	Light	1.820	1.000	Large	unf	unf	-	unf	fav	fav	fav	unf	unf	fav	unf	fav	unf	unf	unf	fav	-	unf	unf	unf	unf	-
	TDB	WL	S4108	0.342	1.000	Dark	0.053	1.000	Small	unf	unf	-	fav	unf	unf	-	fav	unf	unf	fav	fav	unf	unf	unf	unf	fav	unf	fav	unf	-	unf
	TDB	WL	S4764	0.057	1.000	Light	0.355	1.000	Large	unf	unf	-	-	unf	unf	fav	fav	unf	unf	-	fav	unf	unf	unf	unf	fav	-	fav	unf	unf	unf
	TDB	WL	S5238	0.558	1.000	Light	0.451	1.000	Large	-	fav	unf	fav	fav	fav	fav	fav	fav	fav	fav	-	-	fav	fav	-	fav	fav	fav	fav	fav	fav
