# Supplementary material for: Integrating natural variation through GWAS – genetics of drought and flood tolerance in grass pea reveal independent yet interconnected mechanisms
Source: BMC Plant Biol. 2026 Feb 5;26:442. doi: 10.1186/s12870-026-08229-y (PMC12973615; doi:10.1186/s12870-026-08229-y)
Supplement: Supplementary file 5 — Supplementary Material 5. [file 12870_2026_8229_MOESM5_ESM.pdf]

Supplementary Table S4 - Localization of all significantly associated SNP markers in grass pea genome assembly (Vigoroux et al., 2024) and, if marker position was determined, identification of candidate genes within the same location (using the MapMan and Mercator4 v2.0 web tools). If no gene was found, the nomenclature 'Intergenic Region' (IGR) and light grey color were used. If the marker is not currently mapped to the grass pea genome, dark grey color was applied. Functional annotations and protein family obtained from Swiss-Prot and InterPro-UniProt databases. Trait and treatment (Treat) abbreviations according to text.

Marker	Associated with	BestHit e-value	Marker location and position	Within gene?	InterPro-UniProt functional annotation	InterPro Family Ref.	Swissprot functional annotation	Observations
S0002	TDB in ΔWL	4.44E-74	Lschr1:772720103..772724015	g1606	TCP-1/cpn60 chaperonin family	Pfam: PF00118	RuBisCO large subunit-binding protein subunit beta, chloroplastic	
S0018	TDB in WD	4.44E-74	Lschr1:772030773..772043835	g1610	Ubiquitin carboxyl-terminal hydrolase	Pfam: PF00443	Ubiquitin carboxyl-terminal hydrolase 25	
S0020	TDB in WD	4.44E-74						
S0021	TDB in WD	4.44E-74						
S0154	R/S in WL	4.44E-74	Lschr1:627912928..627916929	g2817	Glycosyl transferases group 1	Pfam: PF00534	Granule-bound starch synthase 2, chloroplastic/amyloplastic	
S0179	TDB in ΔWL	9.53E-76	Lschr1:575695889..575697480	g3220	bZIP transcription factor	Pfam: PF00170	ABSCISIC ACID-INSENSITIVE 5-like protein 2	
S0180	TDB in ΔWL	9.53E-76						
S0225	Cab/Ccx in WW	6.1E-25	Lschr6:404151007..404150944	IGR				
S0258	WUE_{869} in WD; WUE_{869} in ΔWD	1.96E-25	Lschr1:149597698..149603797	g4412	WD domain, G-beta repeat	Pfam: PF00400	no annotation	
S0294	A_{869} in WW	1.6E-19	Lschr7:431672278..431672217	IGR				
S0303	WUE_{869} in WD	4.44E-74	Lschr1:85895317..85918705	g4983	YT521-B-like domain	Pfam: PF04146	30-kDa cleavage and polyadenylation specificity factor 30	
S0310	RWC in WL	4.21E-27	Lschr1:101316149..101320765	g4819	no annotation	no annotation	Uncharacterized membrane protein At1g16860	
S0339	RWC in WL	3.89E-87	Lschr1:58390157..58407639	g5249	ABC transporter transmembrane region	Pfam: PF00664	ABC transporter C family member 2	
S0353	Cb in ΔWD	6.5E-126	Lschr1:35068627..35068292	IGR				
S0354	RWC in WL	6.82E-132	Lschr1:35499016..35502501	g5491	B3 DNA binding domain	Pfam: PF02362	Auxin response factor 3	
S0363	WUE_{869} in WD; A_{347} in WW	5.94E-68	Lschr1:22923145..22924060	g5628	Syntaxin	Pfam: PF00804	Syntaxin-124	
S0511	WUE_{869} in WD; WUE_{869} in ΔWD	4.44E-74	Lschr2:459292970..459297169	g8155	HAT (Half-A-TPR) repeat	Pfam: PF02184	no annotation	
S0546	RWC in WL	4.2E-138	Lschr7:307919362..307919089	IGR				
S0558	TDB in WL	6.4E-138	Lschr1:306461540..306461813	IGR				
S0603	RWC in WL	1.23E-74	Lschr2:4563424..4567446	g9427	DHHC palmitoyltransferase	Pfam: PF01529	Probable protein S-acyltransferase 22	
S0644	A_{869} in WW	1.96E-25	Lschr2:547757159..547758239	g6124	SET domain	Pfam: PF00856	Histone-lysine N-methyltransferase ASHR2	
S0676	TDB in ΔWL	2.9E-16	Lschr6:17286130..17286189	IGR				
S0787	WUE_{869} in WD	9.4E-25	Lschr3:664134753..664134690	IGR				
S0809	TDB in WL	4.21E-27	Lschr4:10346754..10347383	g13939.m1	no annotation	no annotation	no annotation	
S0821	Cb in ΔWD	2.06E-72	Lschr3:634869322..634873869	g10663	DUF761-associated sequence motif	Pfam: PF14383	no annotation	
S0822	Cb in ΔWD	2.06E-72						
S0837	SPAD in WD	4.44E-74	Lschr3:611217767..611223852	g10884	KIP1-like protein	Pfam: PF07765	Protein NETWORKED 1B	
S0853	TDB in ΔWD	4.21E-27	Lschr3:592849973..592854243	g11108	Raffinose synthase or seed imbibition protein Sip1	Pfam: PF05691	Probable galactinol--sucrose galactosyltransferase 2	
S0870	Fv/Fo in WW; Fv/Fm in WW	8.98E-126	Lschr3:570738448..570745588	g11301	AAA+ lid domain	Pfam: PF17862	Calmodulin-interacting protein 111	
S0932	RWC in WL	4.33E-76	Lschr3:478790120..478792965	g12119	Protein tyrosine and serine/threonine kinase	Pfam: PF07714	Serine/threonine-protein kinase STY13	
S1051	WUE_{869} in WD; WUE_{869} in ΔWD	2.44E-136	Lschr1:871770402..871774062	g855	Thioredoxin-like [2Fe-2S] ferredoxin	Pfam: PF01257	NADH dehydrogenase [ubiquinone] flavoprotein 2, mitochondrial	
S1073	Cab/Ccx in ΔWD	2.67E-71	Lschr4:639994265..640011172	g17344	alpha/beta-Hydrolases	Superfamily: SSF53474	Embryogenesis-associated protein EMB8	Favourable allele significantly more frequent in Light-seeded and Large-seeded accessions
S1100	A_{869} in WW	4.21E-27	Lschr4:608975094..608978662	g16933	Zn-finger in ubiquitin-hydrolases and other protein	Pfam: PF02148	Ubiquitin C-terminal hydrolase 22	
S1222	RWC in WL	4E-23	Lschr4:493006937..493006874	IGR				
S1252	Fv/Fo in WD; Fv/Fm in WD	1.96E-25	Lschr4:441372715..441374725	g15681	Armadillo/beta-catenin-like repeat	Pfam: PF00514	no annotation	
S1254	PC4 in WD	1.02E-108	Lschr4:439147449..439151107	g15657	Xylanase inhibitor C-terminal	Pfam: PF14541	Aspartyl protease family protein 1	
S1255	PC4 in WD	1.02E-108						
S1286	R/S in WD	4.13E-124	Lschr4:377207828..377209628	g15369	AUX/IAA family	Pfam: PF02309	Auxin-responsive protein IAA9	
S1291	TDB in WD	9.4E-25	Lschr1:15127862..15127925	IGR				
S1312	E_{347} in ΔWL; gs_{347} in ΔWL	1.3E-39	Lschr4:111351305..111354460	g14828	Pectinesterase	Pfam: PF01095	Probable pectinesterase/pectinesterase inhibitor 47	
S1318	E_{347} in ΔWL; gs_{347} in ΔWL	1.3E-39						
S1323	RWC in WW	5.74E-73	Lschr4:97681474..97685721	g14777	Basic region leucine zipper	Pfam: PF07716	Transcription factor TGAL1	Favourable allele significantly more frequent in Light-seeded and Large-seeded accessions
S1361	TDB in ΔWL	1.96E-25	Lschr4:23170358..23170802	g14223	zinc-finger of the FCS-type, C2-C2	Pfam: PF04570	FCS-Like Zinc finger 18	Favourable allele present in more of the selected 'WL tolerant' accessions (than in the 'WL susceptible' ones)
S1389	WUE_{869} in WD	9.2E-134	Lschr7:142653235..142653508	IGR				
S1429	WUE_{869} in WD	4.44E-74	Lschr4:17229192..17236133	g14112	LsmAD domain	Pfam: PF06741	Polyadenylate-binding protein-interacting protein 4	
S1452	RWC in WL	4.21E-27	Lschr4:12813758..12829241	g14004	Exocyst complex component Sec6	Pfam: PF06046	Exocyst complex component SEC6	
S1462	TDB in ΔWD	5.35E-123	Lschr4:54093360..54097062	g14577	Protein kinase domain	Pfam: PF00069	Shaggy-related protein kinase theta	
S1463	TDB in WL	1.7E-132	Lschr5:694020007..694019736	IGR				
S1477	RWC in WL	1.55E-127	Lschr2:505942360..505947981	g7122	no annotation	no annotation	no annotation	
S1513	WUE_{869} in WD; WUE_{869} in ΔWD	UNMAPPED						
S1556	A_{869} in WW	4.21E-27	Lschr5:645574576..645577855	g21818	Prolyl oligopeptidase family	Pfam: PF00326	no annotation	
S1616	Cb in ΔWD	3.43E-75	Lschr5:581265122..581265959	g21243	Plants and Prokaryotes Conserved (PCC) domain	Pfam: PF03479	AT-hook motif nuclear-localized protein 17	
S1668	WUE_{869} in WD	4E-23	Lschr5:538175951..538175888	IGR				
S1708	RWC in WL	1.16E-128	Lschr5:509011641..509014203	g20489	Aldehyde dehydrogenase family	Pfam: PF00171	Aldehyde dehydrogenase family 3 member H1	
S1839	WUE_{869} in WD; WUE_{869} in ΔWD	2.03E-70	Lschr5:410332131..410332698	g19534	Sugar (and other) transporter	Pfam: PF00083	Sugar transport protein 5	
S1840	WUE_{869} in WD; WUE_{869} in ΔWD	2.03E-70						
S1872	TDB in ΔWL	4.21E-27	Lschr5:364104545..364113392	g19206	Protein tyrosine and serine/threonine kinase	Pfam: PF07714	Probable inactive receptor-like protein kinase At3g56050	
S1930	WUE_{869} in WD; WUE_{869} in ΔWD	4.44E-74	Lschr5:48515508..48530430	g18439	Translation initiation factor eIF3 subunit 135	Pfam: PF12807	Clustered mitochondria protein	
S2026	RWC in WL	9.4E-25	Lschr3:20731599..20731536	IGR				
S2102	Cab in WD; Ccx in WD	1.87E-90	Lschr1:877166910..877173278	g782	ABC transporter	Pfam: PF00005	Pleiotropic drug resistance protein 1	
S2133	WUE_{869} in WD	2.08E-67	Lschr6:9433438..9437062	g22613	Proteasome regulatory subunit C-terminal	Pfam: PF08375	Probable 26S proteasome non-ATPase regulatory subunit 3	Favourable allele present in more of the selected 'WD tolerant' accessions (than in the 'WD susceptible' ones)

S2166	TDB in ΔWL	5.74E-73	Lschr6:5998502..6017388	g22480	Exocyst complex component Sec3	Pfam: PF09763	Exocyst complex component SEC3A	
S2242	TDB in WL	5.74E-73	Lschr6:35700977..35710225	g23138	no annotation	no annotation	Zinc finger CCCH domain-containing protein 7	
S2269	WUE$_{869}$ in WD; WUE$_{869}$ in ΔWD	2.6E-23	Lschr7:22696723..22696660	IGR				
S2271	RWC in WL	1.3E-20	Lschr6:518079328..518079265	IGR				
S2299	A$_{869}$ in ΔWL	4.21E-27	Lschr6:467038427..467042185	g24453	TIR domain	Pfam: PF01582	TMV resistance protein N	
S2316	WUE$_{869}$ in WD; WUE$_{869}$ in ΔWD	2.47E-126	Lschr6:449829925..449833539	g24330	Membrane magnesium transporter	Pfam: PF10270	Membrane magnesium transporter	
S2318	WUE$_{869}$ in WD; WUE$_{869}$ in ΔWD	2.47E-126						
S2319	WUE$_{869}$ in WD; WUE$_{869}$ in ΔWD	2.47E-126						
S2369	WUE$_{869}$ in ΔWD	6.1E-25	Lschr6:62241108..62241045	IGR				
S2404	TDB in WD; A$_{869}$ in WW; E$_{869}$ in WW; gs$_{347}$ in WW	1.01E-108	Lschr6:537360727..537363082	g25086	C2 domain	Pfam: PF00168	no annotation	
S2507	RWC in WL	UNMAPPED						
S2565	RWC in WL	UNMAPPED						
S2566	RWC in WL	2.6E-23	Lschr7:659906235..659906172	IGR				Favourable allele significantly more frequent in Light-seeded and Large-seeded accessions
S2616	A$_{869}$ in WD	1.23E-74	Lschr6:653142694..653146772	g26469	WD domain, G-beta repeat	Pfam: PF00400	Elongator complex protein 2	
S2636	WUE$_{347}$ in WL	2.06E-72	Lschr6:669649731..669663431	g26652	HEAT-like repeat	Pfam: PF13513	Transportin-1	
S2661	Cab in WW	6.1E-25	Lschr6:680058130..680058193	IGR				
S2672	WUE$_{347}$ in WL	1.3E-102	Lschr6:682285907..682290083	g26878	Amidohydrolase family	Pfam: PF01979	no annotation	
S2681	RWC in WL	9.12E-24	Lschr3:707379863..707386104	g9654	EF-hand domain	Pfam: PF17958	Probable serine/threonine protein phosphatase 2A regulatory subunit B''Δ	
S2693	E$_{869}$ in ΔWL	4.21E-27	Lschr7:714311378..714313644	g27034	Galactose oxidase, central domain	Pfam: PF13418	Adagio protein 3	
S2718	RWC in WL	3.13E-62	Lschr7:697921143..697952797	g27235	Alpha amylase, catalytic domain	Pfam: PF00128	Isoamylase 1, chloroplastic	
S2719	RWC in WL	3.13E-62						
S2740	WUE$_{869}$ in WD	4.1E-129	Lschr7:684221463..684225211	g27429	Peptidase M1 N-terminal domain	Pfam: PF17900	Puromycin-sensitive aminopeptidase	
S2742	WUE$_{869}$ in WD; WUE$_{869}$ in ΔWD	9.9E-135						
S2857	SPAD in WD	4.21E-27	Lschr7:619238841..619241490	g28280	Mitochondrial ribosomal subunit protein	Pfam: PF10213	no annotation	
S2872	RWC in WL	9.53E-76	Lschr7:612468762..612473287	g28371	K+ potassium transporter	Pfam: PF02705	Potassium transporter 10	
S2874	RWC in WL	9.53E-76						
S2878	RWC in ΔWL	4.44E-74	Lschr7:611781780..611784138	g28385	Aspartate/ornithine carbamoyltransferase, Asp/Orn binding domain	Pfam: PF00185	Aspartate carbamoyltransferase 3, chloroplastic	
S2977	R/S in WL	4.21E-27	Lschr7:500859595..500873539	g29796	Fructose-bisphosphate aldolase class-I	Pfam: PF00274	Fructose-bisphosphate aldolase 1, chloroplastic (Fragment)	
S2983	RWC in WL	2.4E-56	Lschr2:18253740..18279693	g9314	Cyclophilin type peptidyl-prolyl cis-trans isomerase/CLD	Pfam: PF00160	Peptidyl-prolyl cis-trans isomerase CYP23	
S2996	RWC in WL	4.87E-112	Lschr5:454109699..454112380	g19876	Conserved region of unknown function on GLTSCR protein	Pfam: PF15249	no annotation	
S3020	WUE$_{869}$ in WD	5.38E-118	Lschr7:537697359..537700810	g29369	no annotation	no annotation	Protein CONSERVED ONLY IN THE GREEN LINEAGE 160, chloroplastic	
S3023	E$_{869}$ in ΔWL	1.3E-20	Lschr7:533588794..533588731	IGR				Favourable allele significantly more frequent in Dark-seeded and Small-seeded accessions
S3074	WUE$_{869}$ in WW	0.000000063	Lschr2:447989088..447989135	IGR				
S3107	E$_{869}$ in WL	1.3E-71	Lschr5:7076524..7076680	IGR				
S3240	WUE$_{869}$ in WD	1.9E-14	Lschr1:508252265..508252314	IGR				
S3386	RWC in WL	6.7E-14	Lschr1:709441624..709441576	IGR				
S3395	WUE$_{869}$ in WD	9.4E-25	Lschr4:391861007..391861070	IGR				
S3489	RWC in WD; TDB in WD; TDB in WL	9.4E-25	Lschr4:159162294..159162231	IGR				Multi-tolerance *locus* ? (Associated to TDB in both WD and WL)
S3553	R/S in WD	4.21E-27	Lschr1:898674015..898678373	g517	Oxidoreductase family, C-terminal alpha/beta domain	Pfam: PF02894	no annotation	
S3619	TDB in WL	5.9E-21	Lschr1:37286407..37286344	IGR				
S3623	R/S in WL	UNMAPPED						
S3657	WUE$_{869}$ in WD	1.96E-25	Lschr4:644232887..644239857	g17394	DENN (AEX-3) domain	Pfam: PF02141	no annotation	
S3670	RWC in WL	9.4E-25	Lschr1:243067873..243067936	IGR				
S3774	WUE$_{869}$ in WW	9.12E-24	Lschr6:24503242..24510254	g22967	Glycosyl transferase family 1	Pfam: PF13528	L-arabinokinase	
S3852	WUE$_{869}$ in WD	4E-23	Lschr1:760052680..760052617	IGR				
S3871	TDB in WD; TDB in WL	9.4E-25	Lschr4:325572479..325572416	IGR				Multi-tolerance *locus* ? (Associated to TDB in both WD and WL)
S3984	RWC in WL	6.1E-25	Lschr6:572884688..572884751	IGR				
S4026	TDB in WD	UNMAPPED						
S4108	TDB in WL	UNMAPPED						
S4163	E$_{869}$ in ΔWL; E$_{347}$ in ΔWL	UNMAPPED						
S4194	Cab in WD	6.1E-25	Lschr6:434205466..434205403	IGR				
S4328	WUE$_{347}$ in WL; PC3 in WL	1.96E-25	Lschr3:679926871..679929017	g10076	Pectinesterase	Pfam: PF01095	Probable pectinesterase/pectinesterase inhibitor 41	Favourable allele significantly more frequent in Large-seeded accessions, and in more of the selected 'WL tolerant' accessions (than in the 'WL susceptible' ones)
S4424	RWC in WL	1.3E-20	Lschr7:111945314..111945372	IGR				Favourable allele significantly more frequent in Dark-seeded and Small-seeded accessions
S4696	RWC in WL	UNMAPPED						
S4764	TDB in WL	UNMAPPED						
S4766	RWC in WL	UNMAPPED						
S4883	RWC in WL	4.21E-27	Lschr4:665714520..665721829	g17660	BOP1NT (NUC169) domain	Pfam: PF08145	Ribosome biogenesis protein BOP1 homolog	
S4903	RWC in WL	4E-23	Lschr5:343933951..343933888	IGR				
S5038	WUE$_{869}$ in WD	UNMAPPED						
S5235	WUE$_{869}$ in WD, WUE$_{869}$ in ΔWD	2.06E-72	Lschr4:681162821..681167002	g17909	PHD-finger	Pfam: PF00628	PHD finger protein Alfin1	
S5238	TDB in WL	5.1E-77	Lschr6:377960221..377960058	IGR				
S5269	R/S in WD	1.6E-133	Lschr1:307231254..307231542	IGR				
S5270	R/S in WD; R/S in ΔWD	1.6E-133	Lschr1:307231254..307231542	IGR				
S5360	RWC in ΔWD	4.98E-82	Lschr1:726007218..726024085	g1966	SET domain	Pfam: PF00856	Histone-lysine N-methyltransferase ASHR3	

S5446	TDB in ΔWD	1.8E-136	Lschr7:315609914..315609641	IGR				
S5474	RWC in WD; RWC in ΔWD	2.1E-111	Lschr5:94452026..94451802	IGR				
S5478	WUE_{869} in WD; WUE_{869} in ΔWD	5E-124	Lschr6:190051707..190051959	IGR				
S5567	RWC in WL	1.4E-138	Lschr7:152760210..152759898	IGR				QTL explained high portion (23.41%) of trait phenotypic variance
S5574	RWC in WL	1.4E-138	Lschr7:152760210..152759898	IGR				QTL explained high portion (19.33%) of trait phenotypic variance
